# Nernst–Planck–Gaussian finite element modelling of Ca^2+^ electrodiffusion in amphibian striated muscle transverse tubule–sarcoplasmic reticular triadic junctional domains

**DOI:** 10.3389/fphys.2024.1468333

**Published:** 2024-12-05

**Authors:** Marco D. Rodríguez, Joshua A. Morris, Oliver J. Bardsley, Hugh R. Matthews, Christopher L.-H. Huang

**Affiliations:** ^1^ Physiological Laboratory, University of Cambridge, Cambridge, United Kingdom; ^2^ Department of Veterinary Medicine, University of Cambridge, Cambridge, United Kingdom; ^3^ Department of Biochemistry, University of Cambridge, Cambridge, United Kingdom

**Keywords:** skeletal muscle, excitation–contraction coupling, triad junction, calcium microdomains, Nernst–Planck equation, electrodiffusion

## Abstract

**Introduction:**

Intracellular Ca^2+^ signalling regulates membrane permeabilities, enzyme activity, and gene transcription amongst other functions. Large transmembrane Ca^2+^ electrochemical gradients and low diffusibility between cell compartments potentially generate short-lived, localised, high-[Ca^2+^] microdomains. The highest concentration domains likely form between closely apposed membranes, as at amphibian skeletal muscle transverse tubule–sarcoplasmic reticular (T-SR, triad) junctions.

**Materials and methods:**

Finite element computational analysis characterised the formation and steady state and kinetic properties of the Ca^2+^ microdomains using established empirical physiological and anatomical values. It progressively incorporated Fick diffusion and Nernst–Planck electrodiffusion gradients, K^+^, Cl^−^, and Donnan protein, and calmodulin (CaM)-mediated Ca^2+^ buffering. It solved for temporal–spatial patterns of free and buffered Ca^2+^, Gaussian charge differences, and membrane potential changes, following Ca^2+^ release into the T-SR junction.

**Results:**

Computational runs using established low and high Ca^2+^ diffusibility (*D*_Ca2+_) limits both showed that voltages arising from intracytosolic total [Ca^2+^] gradients and the counterions little affected microdomain formation, although elevated *D*_Ca2+_ reduced attained [Ca^2+^] and facilitated its kinetics. Contrastingly, adopting known cytosolic CaM concentrations and CaM-Ca^2+^ affinities markedly increased steady-state free ([Ca^2+^]_free_) and total ([Ca^2+^]), albeit slowing microdomain formation, all to extents reduced by high *D*_Ca2+_. However, both low and high *D*_Ca2+_ yielded predictions of similar, physiologically effective, [Ca^2+^-CaM]. This Ca^2+^ trapping by the relatively immobile CaM particularly increased [Ca^2+^] at the junction centre. [Ca^2+^]_free_, [Ca^2+^-CaM], [Ca^2+^], and microdomain kinetics all depended on both CaM-Ca^2+^ affinity and *D*_Ca2+._ These changes accompanied only small Gaussian (∼6 mV) and surface charge (∼1 mV) effects on tubular transmembrane potential at either *D*_Ca2+_.

**Conclusion:**

These physical predictions of T-SR Ca^2+^ microdomain formation and properties are compatible with the microdomain roles in Ca^2+^ and Ca^2+^-CaM-mediated signalling but limited the effects on tubular transmembrane potentials. CaM emerges as a potential major regulator of both the kinetics and the extent of microdomain formation. These possible cellular Ca^2+^ signalling roles are discussed in relation to possible feedback modulation processes sensitive to the μM domain but not nM bulk cytosolic, [Ca^2+^]_free_, and [Ca^2+^-CaM], including ryanodine receptor-mediated SR Ca^2+^ release; Na^+^, K^+^, and Cl^−^ channel-mediated membrane excitation and stabilisation; and Na^+^/Ca^2+^ exchange transport.

## 1 Introduction

Intracellular Ca^2+^ is key to cellular signalling, regulating membrane permeabilities, enzyme activity, and gene transcription, typically at high nM, and triggering excitation–contraction coupling and apoptosis at µM bulk cytosolic concentrations. In addition to the extracellular space and cytosol, it occurs within membrane-bound organelles, including the endoplasmic reticulum (ER) and mitochondria. Its concentrations in the different compartments are tightly regulated at markedly different levels (e.g., extracellular space, ∼3 mM, and cytosol, ∼50 nM). It is also heavily buffered, the latter forming an important source of [Ca^2+^]_i_ regulation: over 99% of cytoplasmic Ca^2+^ is protein-bound. Ca^2+^ itself is poorly mobile, diffusing 100-fold slower than K^+^ or Cl^−^. The resulting large transmembrane gradients driving Ca^2+^ fluxes and its buffering and poor diffusibility predispose to the generation of Ca^2+^ *microdomains* at the mouth of the translocating Ca^2+^ channels. These are spatially and temporally restricted “clouds” of high [Ca^2+^] potentially involved in local highly specific cellular regulatory actions.

Microdomains are important in cellular signalling. Thus, neuronal L- but not N- or P/Q-Cav-mediated extracellular Ca^2+^ entry triggers CREB Ser^133^ phosphorylation (Dolmetsch et al., 2001) through specific effectors within microdomains specifically around L-Cavs in the L-Cav “channelosome.” These are insensitive to microdomains around other channels because [Ca^2+^] then decays sharply away from L-Cavs. Furthermore, cellular regions with closely apposed membranes further restricting Ca^2+^ diffusion, promoting microdomain formation, are common and important. Thus, plasma membrane (PM)–endoplasmic reticular (ER) junctions formed during T-lymphocyte activation coordinate Ca^2+^ entry; failure of this microdomain formation leads to severe combined immunodeficiency (Feske et al., 2006). Similar membrane-restricted compartments enabling Ca^2+^ accumulation occur in a wide range of cells during store-operated Ca^2+^ entry.

Finally, regarding skeletal and cardiac muscle, triad and dyad, transverse tubule–sarcoplasmic reticular (T-SR) junctions, those in amphibian skeletal muscle, have been anatomically characterised in quantitative detail by electron microscopic methods (Franzini-Armstrong, 1970; 1973). Propagated surface membrane action potentials are conducted into T-tubular membranes invaginating deep within the cell. Pairs of sarcoplasmic reticular (SR) terminal cisternae come into proximity with T-tubular membranes at regular intervals. These form triads, each comprising two SR cisternae and one T-tubule. The T-SR gaps are extremely narrow (12 nm), permitting contained tubular dihydropyridine receptors (DHPRs) and bridging SR ryanodine receptor (RyR) membrane proteins to allosterically interact (Martin et al., 2003; Usher-Smith et al., 2007). Tubular depolarisation triggers DHPR conformational changes, which, in turn, activates the RyR gating SR Ca^2+^ efflux into the tight T-SR space, potentially forming Ca^2+^ microdomains, whose subsequent flux into the remaining cytosol mediates excitation–contraction coupling (ECC) (Huang et al., 2011).

Even in relatively well anatomically characterised skeletal muscle T-SR junctions, such Ca^2+^ microdomains are difficult to study experimentally. They are small, confined between membranes, dispersed over the cell anatomy, and release Ca^2+^ in smaller quantities than those in other signalling events. The [Ca^2+^] changes involved are low compared to other cytosolic ion concentrations, necessitating measurement techniques highly specific to Ca^2+^. However, many of these are unsuited to study the microdomain, as opposed to bulk cytosolic [Ca^2+^]. Many fluorescent indicator-based methods use high-affinity Ca^2+^ buffers such as Mag-Fluo-4 and GCaMP that themselves perturb local Ca^2+^ and have too low a temporal resolution (Despa et al., 2014; Sanchez et al., 2021; Saucerman and Bers, 2012). Some electrophysiological methods have the necessary temporal but not the necessary spatial resolution. For example, Ca^2+^-dependent Cl^−^ current measurements similarly only measure cell-wide Ca^2+^ signalling events.

Alternative theoretical modelling approaches used detailed quantitative characterisations of the cellular anatomy of the skeletal muscle to develop geometrical models of the T-SR junction, permitting mathematical modelling of their regional Ca^2+^ diffusion properties (Bardsley et al., 2021). This initial study demonstrated the formation of Ca^2+^ microdomains of potential physiological importance, resulting in [Ca^2+^] attaining concentrations of ∼20 µM at the microdomain centre. However, it was confined to simple diffusion equations applied to free Ca^2+^ diffusion in the absence of other relevant physiological factors. This prompted the present more quantitative and physiologically realistic approach. We thus now incorporated contributions from charge build-ups within the domain, effects of other ions and major Ca^2+^ buffers. We further investigated for consequent effects on transmembrane potentials and membrane surface charge.

## 2 Theory

### 2.1 Overview of the approach

The study first generated a MATLAB model simulating Ca^2+^ entry and diffusion through and out of the T-SR junction space, adopting parameters used in the existing Ca^2+^ diffusional model in order to permit comparisons between the results of the two studies (Bardsley et al., 2021). It then successively incorporated contributions from cytosolic K^+^ and Cl^−^, Donnan protein, and Ca^2+^ buffers and then additionally examined the effects of the consequent [Ca^2+^] patterns on the membrane potential and surface charge, providing a physiologically realistic analysis of T-SR junctional Ca^2+^ domain formation and properties.

**FIGURE 1:**
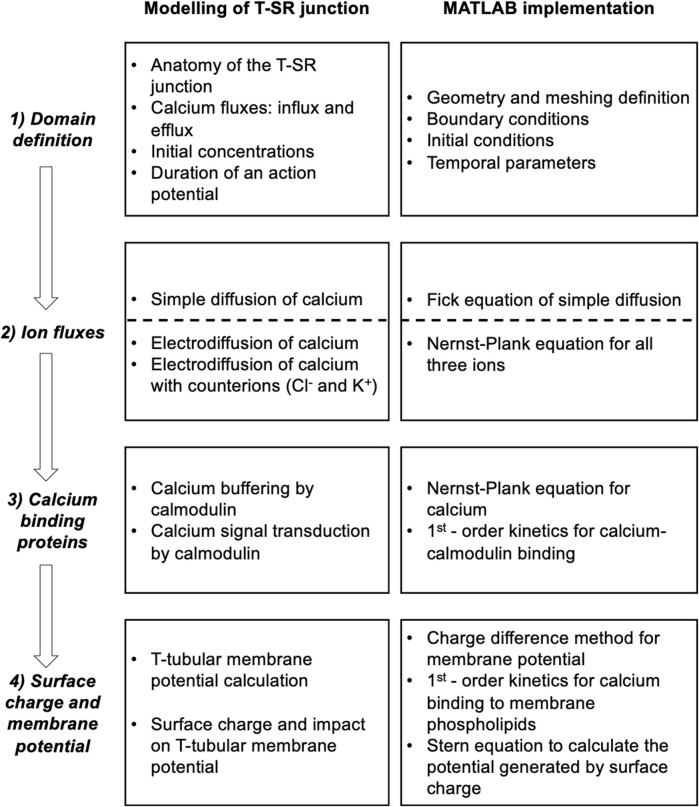
Development and implementation of the T-SR junctional domain model. Successive modelling (left) and implementation steps (right) from (1) definition of the T-SR junction domain model, followed by the progressive implementation of (2) diffusive and electrodiffusive counterion ion fluxes, (3) Ca^2+^ buffers within the domain, and (4) charge difference and surface charge effects on cell membrane potentials.

This analysis involved four main anatomical or computational steps (Figure 1, columns 1 and 2): (1) the domain was defined using previously characterised and adopted dimensions of the junction, cytosolic ion concentrations, and expected fluxes and (2) the equations defining ion behaviour were applied to generate a functional model, including those defining the possible roles of counterions. This analysis of *inorganic ion fluxes* then (i) added electrodiffusion to the previous purely diffusive analyses that had described free Ca^2+^ fluxes and their contribution to microdomain formation. It next (ii) superimposed electrodiffusive flux contributions of other major *in vivo* inorganic K^+^ and Cl^−^, as well as Donnan proteins. This explored the extent to which (i) charge build-up from Ca^2+^ release into a restricted T-SR space generated additional forces for microdomain dissipation, which could reduce accumulated Ca^2+^. Contrastingly, (ii) the resulting fluxes of the highly mobile Cl^−^ and K^+^ counterions could dissipate this charge build-up, reducing the electric potentials generated by Ca^2+^ accumulation, permitting increased maximal [Ca^2+^].

Modelling was then extended, exploring contributions of biological molecules including cytosolic Ca^2+^-binding proteins and phospholipids of the enclosing membrane to microdomain formation and properties. These further steps comprised (3) exploring contributions of cytosolic Ca^2+^ buffers to microdomain formation. For example, binding of Ca^2+^ to Ca^2+^-binding proteins, giving rise to a concentration of bound Ca^2+^, [Ca^2+^]_bound_, could further affect the free Ca^2+^ concentrations, [Ca^2+^]_free_, effective Ca^2+^ diffusion rates, and the total T-SR junction cytosolic Ca^2+^ concentration, [Ca^2+^]. Cytosolic Ca^2+^-binding proteins, besides buffering free Ca^2+^, potentially subserve transduction roles, generating physiologically important signals, and (4) examining the consequences of the consequent charge accumulation on the T-tubule membrane potential. Thus, the altered cytosolic [Ca^2+^]_free_ itself could modify tubular and SR membrane potential through both Gaussian effects of the net charge difference and through changes in membrane surface charge densities generated by the free Ca^2+^, with possible consequences for voltage-gated membrane protein function.

This more realistic and complete Ca^2+^ domain description could shed light on the factors affecting microdomain formation and their implications for its contained regulatory and signalling properties. The above steps are discussed in forthcoming sections.

### 2.2 Definition of domain geometry and meshing

**FIGURE 2:**
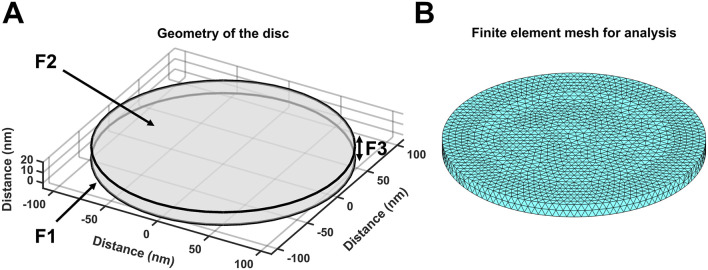
Geometry and mesh representation of a T-SR junction. **(A)** Modelled T-SR junction geometry, where the T-tubular (F1) and SR (F2) membranes are closely apposed. The F3 face opens onto the bulk cytosol of the skeletal myocyte. The junction has diameter *d* = 220 nm in the radial (*XY*) plane, and the membranes are separated by a junctional gap of only *w* = 12 nm in the axial (*Z*) plane. This membrane-enclosed compartment restricts the diffusion of Ca^2+^, allowing high-[Ca^2+^] build-up. **(B)** Finite element meshing of the volume into tetrahedrons of maximal edge length *H*_max_ = 6 nm.

The equations used in the MATLAB PDE Toolbox correspondingly defined the model’s boundary conditions (BCs), simple ion diffusion, ion electrodiffusion, Ca^2+^–calmodulin (CaM) binding, and consequent bulk and surface membrane potentials (Figure 1, column 2). Table 1 summarises (i) previously reported overall geometrical and capacitative properties of amphibian skeletal muscle used to calculate (ii) key sarcomere and tubular membrane surface areas and sarcomere volumes. These were combined with (iii) previously reported electron microscopically measured dimensions to derive a geometrical model of the T-SR junction along previously reported lines (Bardsley et al., 2021). This further made it possible (iv) to quantify the numbers of T-SR junctions required to replicate previously reported Ca^2+^ release fluxes. The resulting formalised representation of a given T-SR junction was a cylinder, with radius *d*/2 = 110 nm and *w* = 12 nm (Figure 2). In Figure 2A, F1 represents the T-tubular membrane, and F2, the SR membrane. The F3 face opens onto the bulk cytosol. Figure 2B shows the subdivision into finite elements (meshing) of the volume for finite element analysis. The Toolbox meshes the volume into tetrahedrons of maximal edge length *H*_max_. Meshing was generated *de novo* on every run, explaining potential inter-run variability. Nevertheless, spatial resolution was set high enough (*H*_max_ ≤ 12 nm) such that inter-run variability was insignificant.

**TABLE 1:** Structural characteristics of amphibian skeletal muscle fibres and transverse tubular–sarcoplasmic reticular (T-SR) junctions.

Name of the variable	Symbol/equation	Value (physiological unit)	Dimension (physiological unit)	Value (SI unit)	Dimension (SI unit)	Source
(i) Muscle fibre geometrical dimensions						
Length of the sarcomere	*l*	3.6	µm	3.65 × 10^−6^	m	Gordon et al. (1966)
Diameter of the fibre	*a*	100	µm	100 × 10^−6^	m	Adrian and Peachey (1973)
Surface membrane-specific capacitance	*C*_s_	1.0	µF/cm^2^	0.01	F/m^2^	Adrian and Peachey (1973)
T-tubular membrane-specific capacitance	*C*_T_	5.0	µF/cm^2^	0.05	F/m^2^	Falk and Fatt (1964)
(ii) Variables derived from muscle fibre geometrical dimensions						
Ratio of T-tubular to surface membrane capacitance	*C*_T_ */C*_s_	5.0		5.0		
Sarcomere surface membrane area	*A*_s_ *= πal*	1,147	µm^2^	1.147 × 10^−9^	m^2^	
Sarcomere tubular membrane area	*A*_T_ *= πalC*_T_ */C*_s_	5,733	µm^2^	5.733 × 10^−9^	m^2^	
Sarcomere volume	ϑ *= πa* ^*2*^ *l/4*	2.87 × 10^4^	µm^3^	2.87 × 10^−14^	m^3^	
(iii) T-SR junction geometrical dimensions						
Proportion of the T-tubular membrane area opposed to the SR	*ξ*	0.3		0.3		Franzini-Armstrong (1970)
Width of the T-SR junction	*w*	12	nm	12 × 10^−9^	m	Franzini-Armstrong (1970)
Diameter of SR terminal cisternae	*d*	220	nm	220 × 10^−9^	m	Franzini-Armstrong (1973), Dulhunty (2006)
(iv) Variables derived from T-SR junction geometrical dimensions						
Area of the SR membrane of the T-SR junction	*πd* ^2^/4	38,013.27	nm^2^	3.801327 × 10^−14^	m^2^	
Area at the edge of the T-SR junction	*πdw*	8,293.804	nm^2^	8.2938	m^2^	
Ratio of the volume of T-SR spaces to that of the whole cell	*ξwA*_T_/ ϑ	7.20 × 10^−4^		7.20 × 10^−4^		
Tubular membrane area abutted by the T-SR junction	*A*_TSR_ = *ξA*_T_	1,720	µm^2^	1.720 × 10^−9^	m^2^	
Total number of T-SR junctions in one sarcomere	4*A*_TSR_/*πd* ^2^	4.5248 × 10^4^		4.5248 × 10^4^		
Total number of T-SR junctions in a unit volume of muscle	*N*_TSR_ = 16*ξC*_T_/*πd* ^2^ *aC*_s_	1.5784 × 10^15^	dm^-3^	1.5784 × 10^18^	m^−3^	

### 2.3 Domain flux boundary and initial modelling conditions

The initial conditions for the start of the modelling process assumed uniform resting ion concentrations equal to their corresponding bulk cytosolic concentrations (Table 2). Neumann BCs were used to describe Ca^2+^ fluxes in and out of the geometry at the edges of the domain, entering and leaving the junction. These set the derivative at each boundary to be equal to a constant. In MATLAB, Neumann BCs are defined as in Equation 1:

$$\vec{n}∙\left(h\nabla c\right)+pc=g,$$ (1)

with $\vec{n}$ the outward unit normal, *h* a constant coefficient, *c* the solution, *p* the transfer coefficient, and *g* the flux density. The analysis first assumed that the entire, *total* Ca^2+^ concentration exclusively comprised *free*, unbuffered Ca^2+^, [Ca^2+^] = [Ca^2+^]_free_. Ca^2+^ diffusive fluxes were then determined by their free diffusion coefficient *D*_Ca2+_. The T-tubular membrane, F1, was modelled as being Ca^2+^-impermeable, with *g* = 0. The SR membrane, F2, was modelled as releasing a constant Ca^2+^ flux throughout the excitation with a constant *g*.

**TABLE 2:** Summary of parameters used in computational modelling.

Definition	Symbol	Value (physiological unit)	Dimensions (physiological unit)	
(i) T-SR junction anatomy (geometry definition)				
T-SR junction radius	*d*/2	110	nm	See Table 1
T-SR distance	*w*	12	nm	See Table 1
(ii) Ion influx and efflux at the edges (boundary conditions)				
Exit length (face F3)	*ϱ*	9.2	nm	Bardsley et al. (2021)
Ca^2+^ flux density (face F2)*	*J*_influx_	3 × 10^−24^	mol.s^−1^.nm^−2^	Bardsley et al. (2021)
(iii) Resting ion concentrations and free diffusion coefficients (initial conditions)				
Resting intracellular [Ca^2+^]	[Ca^2+^]	50	nM	Baylor et al. (2002)
Resting intracellular [K^+^]	[K^+^]	142	mM	Balog and Fitts (1996)
Resting intracellular [Cl^−^]	[Cl^−^]	3	mM	Vaughan-Jones (1982)
Ca^2+^ diffusion coefficient (free)	$D_{Ca2+}$	4 × 10^7^ (low limit) 7 × 10^8^ (high limit)	nm^2^/s	Baylor et al. (2002), Baylor and Hollingworth (1998), Kushmerick and Podolsky (1969), and al-Baldawi Abercrombie (1995)
K^+^ diffusion coefficient	*D*_K+_	2.0 × 10^9^	nm^2^/s	Dubina et al. (2013)
Cl^−^ diffusion coefficient	$D_{Cl-}$	2.0 × 10^9^	nm^2^/s	Passiniemi (1983)
Average diffusion coefficient of soluble cytosolic proteins (Donnan protein)	$D_{Donnan}$	1 × 10^7^	nm^2^/s	Lipkow and Odde (2008)
(iv) Duration and temporal resolution of the simulation				
Duration of the simulation		5 × 10^−4^	s	
Number of time points sampled during the simulation		1,000		
(v) Spatial resolution of finite elements				
Maximal permitted length of the mesh edge	*H*_max_	6	nm	

*value corresponds to a voltage step of the T-tubular membrane to the test voltage of *E* = 0 mV.

We adopted simple, standardised excitation parameters of Ca^2+^ flux through F2 corresponding to full activation at a test voltage of 0 mV. This Ca^2+^ release influx density at each individual T-SR junction, *J*_influx,_ was calculated from previously reported experimental overall initial rates of SR Ca^2+^ release, d[Ca^2+^]/d*t* (Kovacs et al., 1983). This yields the Ca^2+^ influx, $\Phi_{\text{influx}}=$ $\left\{\frac{d\left[{\text{Ca}}^{2+}\right]}{dt}\right\}\vartheta$ , into the sarcomere cytosolic volume *ϑ*. A muscle of sarcomere length *l*, diameter *a*, surface and tubular capacitances of unit surface area, *C*_s_ and *C*_T_, respectively, has a sarcomere tubular membrane area of *A*_T_ *=* (*C*_T_ */C*_s_)π*al.* A proportion ξ of the T-tubular membrane area apposed to triad junctions results in a tubular membrane area abutted by T-SR junctions, $\xi A_{T}$ . The flux density into each T-SR junction is then (Table 1)

$$J_{\text{influx}}=\frac{\left\{\frac{d\left[{Ca}^{2+}\right]}{dt}\right\}\vartheta }{\;\xi A_{T}}.$$ (2)

The Ca^2+^ efflux through the face F3 opening onto the bulk cytosol, $J_{\text{efflux}},$ was modelled with the equation describing its linear dependence on [Ca^2+^] at the edge of the T-SR junction. Here, the previously determined constant exit length, *ϱ* = 9.2 nm (Bardsley et al., 2021), quantified Ca^2+^ diffusion into a well-stirred cytosol, continuously resequestered into the SR by the sarcoplasmic reticular Ca^2+^ (SERCA) pump, leading to steady-state cytosolic–SR flux conservation.

$$g=J_{\text{efflux}}=-\frac{D_{Ca2+}{\left[{Ca}^{2+}\right]}^{edge}}{\varrho }.$$ (3)

### 2.4 Diffusive and electrodiffusive fluxes

The partial differential equations (PDEs) exploring T-SR junction ion fluxes and accumulation were adapted to a format solvable by the MATLAB PDE Toolbox:

$$\lambda_{1}\frac{\partial^{2}c}{\partial t^{2}}+\lambda_{2}\frac{\partial c}{\partial t}-\nabla .\left(\lambda_{3}\nabla c\right)+\lambda_{4}c-\lambda_{5}=0.$$ (4)

The concentration term, *c*, of each diffusible species, *j*, is a function of both radial position, *r*, within the T-SR junction and time *t*, following initiation of ion influx, *c*_*j*_ = *ψ*(*r*, *t*). The coefficients $\lambda_{1}$ – $\lambda_{5}$ are functions of the location (*x*, *y*, and *z*) and can also be functions of the solution *c* or its gradient, at time *t*. Applying the product rule (Equation 5),

$$\nabla .\left(\lambda_{3}\nabla c\right)=\nabla \lambda_{3}.\nabla c+\lambda_{3}\nabla^{2}c.$$ (5)

Given that $\lambda_{3}$ is uniform, $\nabla \lambda_{3}=0$ , giving Equation 6:

$$\nabla .\left(\lambda_{3}\nabla c\right)=\lambda_{3}\nabla^{2}c.$$ (6)

These simplify Equation 4 to give Equation 7:

$$\lambda_{1}\frac{\partial^{2}c}{\partial t^{2}}+\lambda_{2}\frac{\partial c}{\partial t}-\lambda_{3}\nabla^{2}c+\lambda_{4}c-\lambda_{5}=0.$$ (7)

The physical constants used in the following computational solution of the equations adopted standard physically accepted symbols and values: Faraday’s constant, *F* = 96,485.309 C/mol; gas constant, *R* = 8.314511 J/(K.mol); elementary charge, *e =* 1.60217 × 10^−19^ C; free space permittivity, *ε*_0_ = 8.854187817 × 10^−21^ F/nm; cytoplasmic relative permittivity, ε_c_ = 80 (Spencer and Morgan, 2020); and Avogadro’s number, *N*_A_ = 6.0221367 × 10^23^/mol.

Of the adopted flux equations, first, the Fick equation models diffusive fluxes in response to concentration gradients. For diffusion in three dimensions,

$$\frac{\partial c}{\partial t}=D\nabla^{2}c,$$ (8)

with $\frac{\partial c}{\partial t}$ being the rate of concentration change (in mol.m^-3^.s^−1^), *D* the diffusion coefficient (in m^2^.s^−1^), and *c* the concentration (in mol.m^−3^). Comparing terms in Equations 4, 8 then yields the values of each coefficient as summarized in Equation 9:

$$\begin{array}{l} \lambda_{1}=0\\ \;\lambda_{2}=1\\ \;\;\lambda_{3}=D\\ \;\lambda_{4}=0\\ \;\;\lambda_{5}=0. \end{array}$$ (9)

Second, the Nernst–Plank equation (Equation 10) models electrodiffusive fluxes driven by both concentration and voltage terms:

$$\frac{\partial c}{\partial t}=\nabla .D\left[\nabla c+\frac{zF}{RT}c\left(\nabla \varphi \right)\right],$$ (10)

with φ being the electric potential (in V), *T* = 310.15 K (37°C) the temperature (in K), and *z* the valence of the ion. Applying the product rule,

$$\frac{\partial c}{\partial t}-\nabla .\left(D\nabla c\right)-D\frac{zF}{RT}\left(c\nabla^{2}\varphi \right)-D\frac{zF}{RT}\left(\nabla c.\nabla \varphi \right)=0.$$ (11)

Equation 11 is in both concentration *c* and electric potential φ . Following a previous approach, $\nabla^{2}\varphi$ and ∇φ can both be expressed as functions of *c* (Morris et al., 2024). First, expressing the Nernst–Planck equation for separate participating ions *j*:

$$\frac{\partial c_{j}}{\partial t}-D_{j}\nabla^{2}c_{j}-D_{j}\frac{z_{j}F}{RT}c_{j}\nabla^{2}\varphi -D_{j}\frac{z_{j}F}{RT}\nabla \varphi .\nabla c_{j}=0.$$ (12)

Second, the Poisson equation expresses $\nabla^{2}\varphi$ as a function of *c* (Equation 13) (Gabriel et al., 1996):

$$\nabla^{2}\varphi =-\frac{e}{\varepsilon_{0}\varepsilon_{c}}\left[\Sigma z_{j}c_{j}\right].$$ (13)

Third, ∇φ can be expressed as a function of *c*. Charge differences resulting from Ca^2+^ release into the T-SR junction generate time- and space-dependent changes in the transmembrane potential, *∆V.* Gauss’s flux theorem gives the transmembrane electric flux $∆\Phi_{Ê}$ , resulting from net charge δ *q* enclosed in a medium of relative permittivity $\varepsilon_{c}$ as a function of the corresponding electric field, *Ê*, and the total (tubular and SR) membrane surface area, *S* (Lide, 2004):

$$∆\Phi_{Ê}=∯∆Ê.dS=\frac{\delta q}{\varepsilon_{0}\varepsilon_{c}}.$$ (14)

Since the T-SR space is flat and thin, its radius (*d*/2) greatly exceeds its width *w*, and so, *w* << *d*. Accordingly, the surface area of the rim of the T-SR junction, F3, is much smaller than the surface area of the enclosing F1 and F2 membranes, π*dw* << π*d* ^*2*^ */4*, within which time- and space-dependent transmembrane T-tubular and SR potential changes *∆V* tend to φ . We can define the T-SR junction as comprising a series of coaxial concentric annuli of radius *r*, each of area δ*S*/2 and, therefore, of volume $\frac{w\delta S}{2}$ . Equation 15 gives the charge concentration [*q*] within an annulus:

$$\left[q\right]=F\left[\Sigma z_{j}c_{j}\right].$$ (15)

So, the quantity of charge, *δq*, within a given annulus is

$$\delta q=\frac{wF\delta S\left[\Sigma z_{j}c_{j}\right]}{2}.$$ (16)

As the thickness δr of each annulus is reduced such that δr→0 , neighbouring annuli tend towards equal *δS*, each with equal [*q*], such that the electric flux between neighbouring annuli tends to be 0, mostly traversing their flanking T and SR membranes. For membranes of thickness ζ , $∆Ê=\frac{\varphi }{\zeta }$ (Goldman, 1943) such that

$$∆\Phi_{Ê}=∯∆Ê.dS=\frac{\varphi }{\zeta }\delta S.$$ (17)

Since membrane capacitance $C_{m}=\frac{\varepsilon_{0}\varepsilon_{c}}{\zeta }$ , combining Equations 14, 17 yields

$$\varphi =\frac{\delta q}{C_{m}\delta S}.$$ (18)

Finally, substituting Equation 16 into Equation 18 yields

$$\varphi =\frac{Fw\left[\Sigma z_{j}c_{j}\right]}{{2C}_{m}}.$$ (19)

Hence,

$$\nabla \varphi =\nabla \left(\frac{Fw\left[\Sigma z_{j}c_{j}\right]}{{2C}_{m}}\right)=\frac{Fw}{{2C}_{m}}\nabla \left[\Sigma z_{j}c_{j}\right].$$ (20)

Combining Equations 12, 20 yields

$$\frac{\partial c_{j}}{\partial t}-D_{j}\nabla^{2}c_{j}+D_{j}\frac{z_{j}Fe}{RT\varepsilon_{0}\varepsilon_{c}}c_{j}\left[\Sigma z_{j}c_{j}\right]-D_{j}\frac{z_{j}F^{2}w}{RT{2C}_{m}}\nabla \left[\Sigma z_{j}c_{j}\right].\nabla c_{j}=0.$$ (21)

Comparing terms for Equations 4, 21 yields the coefficients listed in Equation 22:

$$\begin{array}{c} \;\lambda_{1}=0\\ \;\lambda_{2}=1\\ \;\lambda_{3}=D\\ \;\lambda_{4}=D_{j}\frac{z_{j}Fe}{RT\varepsilon_{0}\varepsilon_{c}}\left[\Sigma z_{j}c_{j}\right]\\ \;\lambda_{5}=D_{j}\frac{z_{j}F^{2}w}{RT{2C}_{m}}\nabla \left[\Sigma z_{j}c_{j}\right].\nabla c_{j}. \end{array}$$ (22)

Nernst–Planck modelling was applied to electrodiffusion first of [Ca^2+^], subsequently adding contributions of [K^+^] and [Cl^−^]. In both cases, it additionally included soluble, negatively charged, intracellular membrane-impermeable Donnan proteins at concentrations [Donnan], resulting in bulk T-SR cytosolic electroneutrality at time *t* = 0 (Table 2).

### 2.5 Modelling intradomain Ca^2+^ buffers

The presence of CaM was next modelled as having a uniform concentration throughout the T-SR junctional region. Assuming a finite pool of Ca^2+^ and CaM reversibly binding to produce Ca^2+^-CaM, the *total* Ca^2+^ concentration, [Ca^2+^] = [Ca^2+^]_total_, now comprises contributions from free, [Ca^2+^]_free_, and bound Ca^2+^, [Ca^2+^]_bound_:

$$\left[{\text{Ca}}^{2+}\right]={\left[{\text{Ca}}^{2+}\right]}_{\text{free}}+{\left[{\text{Ca}}^{2+}\right]}_{\text{bound}}.$$ (23)

Correspondingly, the total CaM concentration [CaM] comprises free, [CaM]_free_, and bound [CaM-Ca^2+^] components:

$$\left[\text{CaM}\right]={\left[\text{CaM}\right]}_{\text{free}}+\left[\text{CaM}-{\text{Ca}}^{2+}\right].$$ (24)

At every step taken by the solver, for each node of the mesh, [Ca^2+^-CaM] is approximated by

$$K_{d}=\frac{{\left[{Ca}^{2+}\right]}_{free}\;{\left[CaM\right]}_{free}}{\left[CaM-{Ca}^{2+}\right]}.$$ (25)

Expressing dissociation constant *K*_*d*_, $\left[{Ca}^{2+}\right]$ , $\left[CaM\right]$ , and $\left[CaM-{Ca}^{2+}\right]$ concentration terms in M and combining Equations 23 – 25

$$K_{d}=\frac{\left(\left[{Ca}^{2+}\right]-\left[{CaM-Ca}^{2+}\right]\right)\left(\left[CaM\right]-\left[CaM-{Ca}^{2+}\right]\right)}{\left[CaM-{Ca}^{2+}\right]}.$$ (26)

Equations 25, 26 derive from the definition of $K_{d},{\;K}_{d}=\frac{{\left[A\right]}_{free}\times {\left[B\right]}_{free}}{\left[AB\right]}$ , which assumes a system at equilibrium. Nevertheless, the first-order kinetics of Ca^2+^-CaM binding are much faster than any change in the remaining modelling process. Thus, in the R-state, which occurs at high [Ca^2+^], its rate constant is $k_{on}=3\times 10^{10}$ M^−1^.s^−1^ (Bertini et al., 1994; Faas et al., 2011). Even within each computation time step of $\frac{5\times 10^{-4}}{1000}=$ 0.5 µs, up to 72% of the total Ca^2+^ would have reached equilibrium binding. For example, at [Ca^2+^]_total_ = 10 μM, [Ca^2+^-CaM] could increase by 7 µM every 0.5 µs. In any case, the simulations involving CaM reach a steady state, where the binding kinetics are not relevant.

The overall Ca^2+^ diffusive fluxes are now determined by an effective diffusion coefficient $D_{Ca2+}^{*}$ , reflecting a mean of the free Ca^2+^ and CaM-Ca^2+^ diffusion coefficients, *D*_Ca2+_ and *D*_CaM_, respectively, weighted by their respective concentrations, rather than simply the free diffusion coefficient *D*_Ca2+_:

$$D_{Ca2+}^{*}=\frac{{\;D}_{Ca2+}{\left[{Ca}^{2+}\right]}_{free}+D_{CaM\;}\left[CaM-{Ca}^{2+}\right]}{\left[{Ca}^{2+}\right]}.$$ (27)

Finally, the exit boundary condition in Equation 3 is revised to

$$J_{\text{efflux}}=-\frac{D_{Ca2+}^{*}{\left[{Ca}^{2+}\right]}^{edge}}{\varrho }.$$ (28)

### 2.6 The net Ca^2+^ accumulation in the T-SR junction

The net rate at which Ca^2+^ is trapped within the T-SR junction whether as [Ca^2+^]_free_ or [Ca^2+^]_bound_ in the presence or absence of CaM can be directly determined from balancing the magnitude of the boundary Ca^2+^ influxes and effluxes. The Ca^2+^ influx density through the SR membrane face F2, *J*_influx_, is constant whether CaM is present or absent. The overall influx rate into a single T-SR junction of diameter *d* (Equation 29) can then be obtained from the flux density into each T-SR junction (Equation 2):

$$\Phi_{\text{influx}}=J_{\text{influx}}\left(\frac{\pi d^{2}}{4}\right).$$ (29)

The Ca^2+^ efflux at the T-SR junctional edge face F3, axial distance *w*, opening onto the bulk cytosol, as a function of time (Equation 30) is determined by the exit boundary condition in Equation 28:

$$\Phi_{\text{efflux}}=\frac{D_{Ca2+}^{*}{\left[{Ca}^{2+}\right]}^{edge},}{\varrho }\left(\pi dw\right).$$ (30)

The net rate of T-SR junction Ca^2+^ accumulation or trapping at any given time τ is then the difference (Equation 31):

$$\Phi_{\text{influx}}-\Phi_{\text{efflux}}.$$ (31)

Accordingly, the mean [Ca^2+^], averaged through the entire T-SR space, at any given time, τ , <[Ca^2+^]>_τ_, is the ratio between the *time* integral between the limits (0, τ ) of the flux difference and the T-SR junction volume $\left(\frac{\pi wd^{2}}{4}\right)$ :

$$\begin{array}{l} <\left[{Ca}^{2+}\right]{>}_{\tau }=\int_{0}^{\tau }\frac{\left(\Phi_{\text{influx}}-\Phi_{\text{efflux}}\right)dt}{\left(\frac{\pi wd^{2}}{4}\right)}=\\ \int_{0}^{\tau }\frac{\left[J_{\text{influx}}\left(\frac{\pi d^{2}}{4}\right)-\frac{D_{Ca2+}^{*}{\left[{Ca}^{2+}\right]}^{edge}}{\varrho }\left(\pi dw\right)\right]dt}{\left(\frac{\pi wd^{2}}{4}\right)}. \end{array}$$ (32)

Correspondingly, <[Ca^2+^]>_τ_ is also obtainable from the *spatial* integral through the cross-sectional area of the T-SR junction of the actual concentrations through the cross-sectional area of the T-SR junction at the end of the same time interval (0, τ). Here, the constant term before the integral is the inverse total volume of the T-SR gap junctional region, and the $\left(2\pi rw.dr\right)$ term is the volume of each annular element over which the integration is performed:

$$<\;\left[{Ca}^{2+}\right]{>}_{\tau }=\frac{4}{{\pi d}^{2}w}\;\int_{0}^{d/2}{\left[{\text{Ca}}^{2+}\right]}_{\tau }^{r}\;2\pi rw.dr=\frac{8}{d^{2}}\;\int_{0}^{d/2}{\left[{\text{Ca}}^{2+}\right]}_{\tau }^{r}\;r.dr.$$ (33)

A comparison of the time and spatial integrals in Equations 32, 33 evaluating <[Ca^2+^]>_τ_ provides tests for overall Ca^2+^ conservation throughout the time interval (0, τ), which is discussed in *Results*.

### 2.7 Effects on transmembrane potentials

[Ca^2+^] and [Ca^2+^]_free_ influence the transmembrane potential change, ∆*V*, in two independent ways. Both in turn could influence the function in voltage-sensitive, including T-tubular membrane, proteins. First, accumulation of charged particles in the T-SR space increases its cytosolic potential *V*_i_, thereby affecting ∆*V*. Each T-SR space annular element has width δr , radius *r*, volume ϑ=2πrwδr , total T and SR membrane area S = 4πrδr , and capacitance of the unit membrane area *C*_m_. Its capacitance of unit volume is then

$$C_{\vartheta }=\frac{C_{m}\times 4\pi r.\delta r}{2\pi rw.\delta r}=\frac{{2C}_{m}}{w}.$$ (34)

Combining Equation 34 with Equation 19 for its contained electric potential φ yields Equation 35:

$$\varphi =\frac{F\left[\Sigma z_{j}c_{j}\right]}{C_{\vartheta }},$$ (35)

This is identical in form to the previously introduced charge difference equation predicting membrane potentials from intracellular electrolyte concentrations independent of assumptions of Nernst equilibrium or steady-state ion fluxes required by the classical Goldman–Hodgkin–Katz equation (Fraser and Huang, 2004; Goldman, 1943). This previous application had made resting potential predictions consistent with established experimental analyses (Mullins and Noda, 1963) of equilibrium ( $\frac{d\varphi }{dt}=0$ ) cellular membrane potential values. However, the charge difference equation was introduced for an entire, completely enclosed, well-stirred intracellular space. The present analysis contrastingly generalises to charged particles moving within an element of a partially enclosed space open at its rim. In addition, their electrostatic flux is directed through the T and SR membrane partially containing the element.

The *change* in φ , ∆ φ , was then obtained from the *change* in charge difference. As only divalent [Ca^2+^] changes significantly,

$$∆\varphi =\frac{2F∆\left[{Ca}^{2+}\right]}{C_{\vartheta }}.$$ (36)

Ca^2+^–protein binding/unbinding does not affect ∆ φ as Equation 37 involves the total charge *difference* within the volume. The latter also accounts for the presence of Donnan proteins, which similarly *do not* contribute to ∆ φ .

Second, membrane phospholipids contain high negative intracellular and extracellular surface-charge densities, σ _i_ and $\sigma_{o}$ . These cause negative surface potential alterations at their intracellular, *i*, and extracellular, *o*, plasma membrane faces. The latter are each consequently flanked by Stern layers, with reductions in the intracellular and extracellular electric potentials ∆*V*_*σ*_(*x*) = ∆*V*_*σi*_(*x*_i_) and ∆*V*_*σ*_(*x*) = ∆*V*_*σo*_(*x*_o_), whose respective dependences on *σ* = σ _i_ and *σ* = $\sigma_{o}$ and distances from the plasma membrane, *x = x*_i_ and *x = x*_o_, are given by the Stern equation (McLaughlin, 1989):

$${∆V}_{\sigma }\left(x\right)=-2\frac{RT}{F}\ln\left(\frac{1+\alpha e^{-x\kappa }}{1-\alpha e^{-x\kappa }}\right),$$ (37)

with $\alpha =-b\kappa +\sqrt{b^{2}\kappa^{2}+1}$ , $b=\frac{2\varepsilon \varepsilon_{0}RT}{F\left|\sigma \right|}$ , σ being the surface charge density (in C.m^-2^), and $\kappa^{-1}$ being the Debye length (in m), providing a measure of how the cytosolic electrolytes “screen” the effects of a static charge.

$$\kappa^{-1}=\left(\frac{\varepsilon_{0}\varepsilon_{c}kT}{\sum_{i}z_{j}\rho_{j}}\right),$$ (38)

with *k* being the Boltzmann constant and $\rho_{j}$ the number of ions per m^3^. At the membrane surface, for which *x* = 0, this gives the surface potential:

$${∆V}_{\sigma }\left(0\right)=-2\frac{RT}{F}\ln\left(\frac{1-b\kappa +\sqrt{b^{2}\kappa^{2}+1}}{1+b\kappa -\sqrt{b^{2}\kappa^{2}+1}}\right).$$ (39)

The effects of this surface charge extend for a few nm from the membrane, a distance comparable to the 12-nm T-SR gap with its membranes on both sides, with potential significance.

**FIGURE 3:**
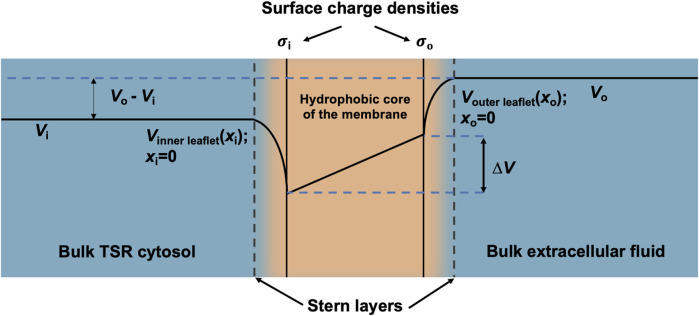
Surface charge density and electric potential in the Stern layers. Typical profile of electric potential across the plasma membrane (not to scale) containing a hydrophobic core and separating the bulk T-SR cytosol and extracellular fluid. In the present analysis, *V*_i_, the bulk cytosolic potential, is subject to change by net charge differences in the intracellular space, whereas *V*_o_, the bulk extracellular potential, is constant. The Stern layer where the electrical potential is lower than expected due to the presence of a negative surface charge on the intracellular and extracellular faces of the plasma membrane ( σ _i_ and $\sigma_{o}$ ) is a few nm thick. The potential within the Stern layer is a function of the surface charge density and distance from the plasma membrane (*x*_i_ and *x*_o_). The surface charges σ _i_ and $\sigma_{o}$ produce voltage changes, ${∆V}_{\sigma i}\left(x_{i}\right)$ and ${∆V}_{\sigma o}\left(x_{o}\right)$ , respectively, that modify the potentials at the inner- and outer-membrane leaflets, *V*_inner leaflet_ (*x*_i_ = 0) and *V*_outer leaflet_ (*x*_o_ = 0), respectively, thereby influencing the transmembrane potential, ∆*V*.

Furthermore, the surface charge modifies the resulting transmembrane potential profile and the consequent actual transmembrane potential ∆*V* (Figure 3). The latter depends on both the overall transmembrane potential decrease between the respective bulk cytosolic, *V*_i_, and extracellular potentials, *V*_o_, and inner- and outer-membrane leaflet surface charge contributions, ∆*V*_*σi*_(0) and ∆*V*_*σo*_(0), respectively, dependent on σ _i_ and $\sigma_{o}$ . Cations bind the underlying negatively charged membrane phospholipid groups, screening their charge and reducing σ . Thus, Ca^2+^ adsorption onto membrane lipids forming the inner membrane leaflet would affect ∆*V* without affecting *V*_i_. This could be assessed using Ca^2+^-binding constants *K*_d_’ for Ca^2+^ association with the membrane phospholipids using Equation 40. This takes the form used for Ca^2+^-CaM binding involving free and Ca^2+^-binding phospholipid-binding sites:

$${K_{d}}^{\prime }=\frac{{\left[{Ca}^{2+}\right]}_{free}\;{\left[phospholipid\;binding\;sites\right]}_{free}}{\left[phospholipid-{Ca}^{2+}\right]}.$$ (40)

## 3 Materials and methods

Table 3 summarises the computational implementation of the equations derived in *Theory*. Modelling was performed on MATLAB (R 2023a, Update 5) using the PDE Toolbox. The equations were solved using a 2020 Mac Book Pro (Apple M1 chip, 8 GB RAM). The modelling process followed the previously described optimised pipeline (Bardsley et al., 2021), involving the following steps (for computational source listings, see Supplementary Material): 1) geometry generation: generating the cylinder representing the T-SR junction according to specified dimensions; 2) meshing of the T-SR junction into finite elements; 3) solving of the PDE(s) using the specified coefficients, initial conditions, and BCs; and 4) plotting: extracting, processing, and plotting the data from the solver. The solutions involving four simultaneous PDEs involving Ca^2+^, K^+^, Cl^−^, and Donnan protein, as opposed to a single PDE involving Ca^2+^ with Neumann BCs, caused excessively long running times. These reflected the computationally expensive exit-length based Neumann BCs describing ion diffusion from the T-SR space into the bulk cytosol at face F3. Such computational cost did not scale linearly with the sequential addition of more equations. For example, at a resolution of *H*_max_ = 12 nm, a one-equation model required 60 s and a two-equation model required 50 min to solve, and models with three and four equations were unsolvable. This was dealt with by utilising a mixed set of F3 boundary conditions. Ca^2+^ diffusion from T-SR into the bulk cytosol used the Neumann BC previously shown to be consistent with experimental measurements (Bardsley et al., 2021). The remaining counterions were modelled with computationally cheaper Dirichlet BCs. Instead of defining a flux orthogonal to the F3 face, this modelled a fixed value for the solution *u* at F3, where, as adopted by the PDE Toolbox, hc=r , where *h* and *r* are space- and time-dependent functions and *c* is the solution. In physiological terms, this assumed that [K^+^] and [Cl^−^] at the junction’s edge were equal to bulk cytosolic concentrations throughout the modelling. This is physiologically accurate as (i) the T-SR volume is negligible compared to the bulk cytosolic volume so that ion fluxes to and from the T-SR do not affect the bulk cytosolic concentration and (ii) K^+^ and Cl^−^ are highly mobile, resulting in K^+^ and Cl^−^, as opposed to Ca^2+^ concentration gradients near the triad rapidly equilibrating.

**TABLE 3:** Equations used at successive modelling stages.

Section	Stage of analysis	Equation
4.2	(A) Domain formation from free-Ca^2+^diffusion into the T-SR space (Bardsley et al., 2021)	Fick diffusion equation
	From Equation 8: $\frac{\partial \left[{Ca}^{2+}\right]}{\partial t}-D_{Ca2+}\left(\nabla^{2}\left[{Ca}^{2+}\right]\right)=0$	[41]
4.3	(B) Domain formation from free-Ca^2+^electrodiffusion into the T-SR space	Nernst–Planck diffusion equation
	From Equation 21: $\frac{\partial \left[{Ca}^{2+}\right]}{\partial t}-D_{Ca2+}\nabla^{2}\left[{Ca}^{2+}\right]-D_{Ca2+}\left(\frac{2Fe}{RT\varepsilon_{0}\varepsilon_{c}}\right)\left[{Ca}^{2+}\right]\left\{\left[Donnan\right]-\right.2\left[{Ca}^{2+}\right]\}-D_{Ca2+}\left(\frac{2F^{2}w}{RT{2C}_{m}}\right)\nabla \left\{2\left[{Ca}^{2+}\right]-\left[Donnan\right]\right\}.\nabla \left[{Ca}^{2+}\right]=0$	[42]
	$\frac{\partial \left[Donnan\right]}{\partial t}-D_{Donnan}\nabla^{2}\left[Donnan\right]+D_{Donnan}\left(\frac{Fe}{RT\varepsilon_{0}\varepsilon_{c}}\right)\left[Donnan\right]\left\{\left[Donnan\right]-2\left[{Ca}^{2+}\right]\right\}+D_{Donnan}\left(\frac{F^{2}w}{RT{2C}_{m}}\right)\nabla \left\{2\left[{Ca}^{2+}\right]-\left[Donnan\right]\right\}.\nabla \left[Donnan\right]=0$	[43]
4.4	(C) Domain formation from free-Ca^2+^electrodiffusion into the T-SR space containing physiological electrolyte concentrations	Nernst–Planck diffusion equation
	From Equation 21: $\frac{\partial \left[{Ca}^{2+}\right]}{\partial t}-D_{Ca2+}\left(\nabla^{2}\left[{Ca}^{2+}\right]\right)-D_{Ca2+}\left(\frac{2Fe}{RT\varepsilon_{0}\varepsilon_{c}}\right)\left[{Ca}^{2+}\right]\left\{\left[{Cl}^{-}\right]+\left[Donnan\right]-2\left[{Ca}^{2+}\right]-\left[K^{+}\right]\right\}-D_{Ca2+}\left(\frac{2F^{2}w}{RT{2C}_{m}}\right)\nabla \left\{2\left[{Ca}^{2+}\right]+\left[K^{+}\right]-\left[{Cl}^{-}\right]-\left[Donnan\right]\right\}.\nabla \left[{Ca}^{2+}\right]=0$	[44]
	$\frac{\partial \left[K^{+}\right]}{\partial t}-D_{K+}\left(\nabla^{2}\left[K^{+}\right]\right)-D_{K+}\left(\frac{Fe}{RT\varepsilon_{0}\varepsilon_{c}}\right)\left[K^{+}\right]\left\{\left[{Cl}^{-}\right]+\left[Donnan\right]-2\left[{Ca}^{2+}\right]-\left[K^{+}\right]\right\}-D_{K+}\left(\frac{F^{2}w}{RT{2C}_{m}}\right)\nabla \left\{2\left[{Ca}^{2+}\right]+\left[K^{+}\right]-\left[{Cl}^{-}\right]-\left[Donnan\right]\right\}.\nabla \left[K^{+}\right]=0$	[45]
	$\frac{\partial \left[{Cl}^{-}\right]}{\partial t}-D_{Cl-}\left(\nabla^{2}\left[{Cl}^{-}\right]\right)+D_{Cl-}\left(\frac{Fe}{RT\varepsilon_{0}\varepsilon_{c}}\right)\left[{Cl}^{-}\right]\left\{\left[{Cl}^{-}\right]+\left[Donnan\right]-2\left[{Ca}^{2+}\right]-\left[K^{+}\right]\right\}+D_{Cl-}\left(\frac{F^{2}w}{RT{2C}_{m}}\right)\nabla \left\{2\left[{Ca}^{2+}\right]+\left[K^{+}\right]-\left[{Cl}^{-}\right]-\left[Donnan\right]\right\}.\nabla \left[{Cl}^{-}\right]=0$	[46]
	$\frac{\partial \left[Donnan\right]}{\partial t}-D_{Donnan}\left(\nabla^{2}\left[Donnan\right]\right)+D_{Donnan}\left(\frac{Fe}{RT\varepsilon_{0}\varepsilon_{c}}\right)\left[Donnan\right]\left\{\left[{Cl}^{-}\right]+\left[Donnan\right]-2\left[{Ca}^{2+}\right]-\left[K^{+}\right]\right\}+D_{Donnan}\left(\frac{F^{2}w}{RT{2C}_{m}}\right)\nabla \left\{2\left[{Ca}^{2+}\right]+\left[K^{+}\right]-\left[{Cl}^{-}\right]-\left[Donnan\right]\right\}.\nabla \left[Donnan\right]=0$	[47]
4.5 and 4.7	(D) Domain formation from Ca^2+^electrodiffusion into a T-SR space containing a Ca^2+^buffer	Nernst–Plank diffusion equation + Ca^2+^buffering
	From Equations 21, 26, 27: $\frac{\partial \left[{Ca}^{2+}\right]}{\partial t}-D_{Ca2+}^{*}\nabla^{2}\left[{Ca}^{2+}\right]-D_{Ca2+}^{*}\left(\frac{2Fe}{RT\varepsilon_{0}\varepsilon_{c}}\right)\left[{Ca}^{2+}\right]\left\{\left[Donnan\right]-2\right.\left[{Ca}^{2+}\right]\}-D_{Ca2+}^{*}\left(\frac{2F^{2}w}{RT{2C}_{m}}\right)\nabla \left\{2\left[{Ca}^{2+}\right]-\left[Donnan\right]\right\}.\nabla \left[{Ca}^{2+}\right]=0$	[48]
	$\frac{\partial \left[Donnan\right]}{\partial t}-D_{Donnan}\nabla^{2}\left[Donnan\right]+D_{Donnan}\left(\frac{Fe}{RT\varepsilon_{0}\varepsilon_{c}}\right)\left[Donnan\right]\left\{\left[Donnan\right]-2\left[{Ca}^{2+}\right]\right\}+D_{Donnan}\left(\frac{F^{2}w}{RT{2C}_{m}}\right)\nabla \left\{2\left[{Ca}^{2+}\right]-\left[Donnan\right]\right\}.\nabla \left[Donnan\right]=0$	[49]
	$K_{d}=\frac{\left(\left[{Ca}^{2+}\right]-\left[{CaM-Ca}^{2+}\right]\right)\left(\left[CaM\right]-\left[CaM-{Ca}^{2+}\right]\right)}{\left[CaM-{Ca}^{2+}\right]}$	[50]
	$D_{Ca2+}^{*}=\frac{{\;D}_{Ca2+}{\left[{Ca}^{2+}\right]}_{free}+D_{CaM\;}\left[CaM-{Ca}^{2+}\right]}{\left[{Ca}^{2+}\right]}$	[51]
4.6	(E) Net Ca^2+^accumulation in the T-SR junction	Conservation equations
	From Equations 28, 32, 33: $J_{\text{efflux}}=-\frac{D_{Ca2+}^{*}{\left[{Ca}^{2+}\right]}^{edge}}{\varrho }$	[52]
	$<\left[{Ca}^{2+}\right]{>}_{\tau }=\int_{0}^{\tau }\frac{{\left[J_{\text{influx}}\left(\frac{\pi d^{2}}{4}\right)-\frac{D_{Ca2+}^{*}{\left[{Ca}^{2+}\right]}^{edge},}{\lambda }\left(\pi dw\right)\right]}_{dt}}{\left(\frac{\pi wd^{2}}{4}\right)}$	[53]
	$<\left[{Ca}^{2+}\right]{>}_{\tau }=\frac{8}{d^{2}}\;\int_{0}^{d/2}\;{\left[{\text{Ca}}^{2+}\right]}_{\tau }^{r}\;r.dr$	[54]
4.8	(F) Effect of the resulting bulk charge difference on the T-tubular membrane potential	Charge difference equation (adapted from Gauss’s law)
	From Equations 35, 36: $\varphi =\frac{F\left[\Sigma z_{j}c_{j}\right]}{C_{\vartheta }}$	[55]
	$∆\varphi =\frac{2F∆\left[{Ca}^{2+}\right]}{C_{\vartheta }}$	[56]
4.8	(G) Effect of Ca^2+^absorption on the inner leaflet of the plasma membrane on the T-tubular membrane	Stern equation
	From Equations 37–39: ${∆V}_{\sigma }\left(0\right)=-2\frac{RT}{F}\ln\left(\frac{1-b\kappa +\sqrt{b^{2}\kappa^{2}+1}}{1+b\kappa -\sqrt{b^{2}\kappa^{2}+1}}\right)$	[57]

## 4 Results

### 4.1 Domain definition and initial conditions

Table 1 summarises *determinations of the domain structure and dimensions* from established electron microscopic anatomical data aligned, therefore ensuring comparability with earlier reports (Bardsley et al., 2021). (i) Reported values of muscle fibre geometrical dimensions of sarcomere length, *l*, fibre diameter, *a*, and surface, *C*_s_, and tubular, *C*_T_, membrane capacitances (Adrian and Peachey, 1973; Falk and Fatt, 1964; Gordon et al., 1966) were used to derive (ii) values of the ratio of T-tubular to surface membrane capacitance, *C*_T_ */C*_s_, and the sarcomere surface membrane area, *A*_s_, tubular membrane area, *A*_T_, and volume, ϑ , using the formulae in column 2. (iii) The previously established T-SR junction geometrical dimensions defining the proportion of the T-tubular membrane area opposed to SR, *ξ* (Franzini-Armstrong, 1970), width of the T-SR junction, *w* (Franzini-Armstrong, 1970), and diameter of the SR terminal cisternae, *d* (Dulhunty, 2006; Franzini-Armstrong, 1973), were then combined with the results in (ii) to derive (iv) values required for determining realistic values for Ca^2+^ fluxes traversing each individual T-SR junction, viz., the area of the SR membrane of the T-SR junction, area at the edge of the T-SR junction, ratio of the total volume of T-SR spaces to that of the whole cell, tubular membrane area abutted by T-SR junctions, and the total number of T-SR junctions in one sarcomere. This yielded the total number of T-SR junctions in a unit volume of muscle, *N*_TSR._

The *initial conditions* listed in Table 2 (i) combine the T-SR gap geometry with details of its meshing for finite element analysis. (ii) They determined the BCs concerning (a) the adopted value of Ca^2+^ influx into each T-SR junction. This was obtained from previous reports, in which a voltage step to test membrane potential, *E* = 0 mV, yielded a maximum rate of [Ca^2+^] increase, d[Ca^2+^]/d*t =* 180 μmol/(dm^3^ s), elevating the bulk peak cytosolic calcium concentration to [Ca^2+^]_max_ = 3.161 μmol/dm^3^ (Kovacs et al., 1983). Combining this with the values given in Table 1 yielded a Ca^2+^ flux density into the T-SR junction, *J*_influx_ = 3.00 × 10^−24^ mol/(nm^2^ s), corresponding to a Ca^2+^ flux into each T-SR junction, *Φ*_influx_ = 1.14 × 10^−19^ mol/s and (b) the exit length *ϱ*, = 9.2 nm. This represented Ca^2+^ diffusion into a well-stirred cytosol, in which it is continuously sequestered by SERCA activity, providing overall flux conservation between cytosolic and SR compartments, derived from previous solutions (Bardsley et al., 2021). They also included (iii) values of adopted counterion concentrations and their diffusion coefficients when these were included in the computational solutions and (iv) duration and temporal and (v) mesh resolution of each run.

The reported values of the biological diffusion coefficient, *D*_Ca_, vary under different conditions over a range of ∼10^7^ nm^2^ s^−1^–10^8^ nm^2^ s^−1^, the lowest empirical values extending to mini-electrode technique measurements of 1.0 × 10^7^ nm^2^ s^−1^ in *Myxicola* axoplasm with intact Ca-sequestering organelles most realistically reflecting bulk cytosolic physiological conditions (al-Baldawi and Abercrombie, 1995). However, the present studies sought to investigate Ca^2+^ diffusion or accumulation in a restricted T-SR space potentially not representative of the whole-cell cytoplasm. Nevertheless, we could perform and compare results from computations using low and high limits for *D*_Ca_, spanning the reported range covering both possibilities.

Adopting the value provided by Baylor and Hollingworth (1998) of *D*_Ca2+_ = 7 × 10^8^ nm^2^ s^−1^ allows for the myoplasmic viscosity being two-fold higher than that of a simple salt solution (Kushmerick and Podolsky, 1969). It also assumes an absence of (1) Ca^2+^-sequestering membrane-bound organelles and (2) cytoplasmic Ca^2+^ buffers. Within the restricted T-SR space, condition (1) is likely fulfilled but not condition (2): even the T-SR-restricted space likely includes a Ca^2+^ buffer additional to CaM that could affect *D*_Ca2+_. Including the entire bulk cytoplasmic Ca^2+^-binding capacity yielded a 50-fold *D*_Ca2+_ reduction, predicting a lower limit of *D*_Ca2+_ = 2.8 × 10^7^ nm^2^ s^−1^ (Kushmerick and Podolsky, 1969). Nevertheless, adopting *D*_Ca2+_ = 7 × 10^8^ nm^2^ s^−1^ provided an upper computational limit that could be compared with findings from a lower *D*_Ca_ limit of 4.0 × 10^7^ nm^2^ s^−1^ derived from the lower end of the range 5–20 × 10^7^ nm^2^ s^−1^ determined from empirical isotope and mini-electrode measurements in ATP-depleted *Myxicola* axoplasm (al-Baldawi and Abercrombie, 1995). It was therefore possible to compare findings and assess the sensitivity of the computational outcomes to *D*_Ca2+_ through these orders of magnitude.

### 4.2 Simple free-Ca^2+^ diffusion modelled using the Fick equation

**FIGURE 4:**
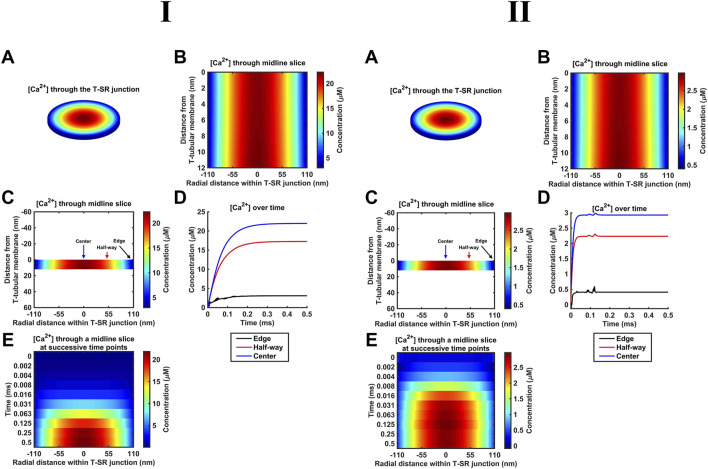
Principal results from a simple Ca^2+^ diffusion model. (A–C) Steady-state T-SR junction [Ca^2+^] profiles, obtained at 0.5 ms. (A,C) conserve the original proportions of the geometry to illustrate the spatial [Ca^2+^] profile, and (B) is distorted for clarity. In (I), note the 22-µM [Ca^2+^] region in the centre of the junction and [Ca^2+^] decreasing to 3 μM at the edge. (D, E) Evolution of [Ca^2+^] concentration over time. Colours of traces in (D) correspond to the markers in (C). A concentration of 20 µM at the centre of the T-SR junction is reached in 0.15 ms. The microdomain adopts and maintains its steady-state profile by 0.25 ms. Studies made at Ca^2+^ diffusion coefficients at low and high limits of *D*_Ca_ = 4 × 10^7^ nm^2^ s^−1^ **(I)** and 7 × 10^8^ nm^2^ s^−1^ **(II)**.

As indicated above (Section 2.1), our diffusional modelling first progressively investigated fluxes of free ions within the T-SR junction, advancing from Fick to Nernst–Planck analysis and from fluxes of Ca^2+^ alone to an inclusion of the remaining intracellular, inorganic, and Donnan ions occurring *in vivo*. We first modelled simple Ca^2+^ diffusion and Ca^2+^ microdomain formation during imposed depolarisation using Fick’s law (implemented as in Table 3 A; Equation 41). Figure 4 confirms the formation of high-[Ca^2+^] microdomains at both the low (Figure 4I) and high (Figure 4I) limiting *D*_Ca_ values to different extents and kinetics. It demonstrates (A–C) radial (A) and axial (B, C) steady-state T-SR junctional [Ca^2+^] profiles reached by 0.5 ms. (A) and (C) conserve the original proportions of the geometry to visualise the spatial [Ca^2+^] profile, while (B) is distorted for clarity. (D) and (E) show the evolution of [Ca^2+^] concentration over time. The colour of traces in (D) corresponds to the markers in (C). At the low *D*_Ca_ limit, a maximum concentration of 22 µM was achieved in the region in the centre of the junction (Figure 4ID). [Ca^2+^] decreased to 3 μM at equilibrium with the bulk cytosolic [Ca^2+^] at the edge. The microdomain was almost fully formed 0.25 ms after the voltage step, maintaining its steady state thereafter (Figure 4IE). At the high *D*_Ca_ limit (Figure 4II), a maximum concentration of 2.9 µM was reached at the centre of the junction (Figure 4IID), and [Ca^2+^] decreased to 0.4 μM at equilibrium with the bulk cytosolic [Ca^2+^] at the edge. The microdomain was fully formed by 0.2 ms after the voltage step (Figure 4IIE).

These results are consistent with the previous findings (Bardsley et al., 2021). The noise in some of the [Ca^2+^] traces (Figure 4D, edge) is attributable to the stiff nature of the Neumann BC used to model Ca^2+^ flux through face F3. The noise could be reduced by increasing the temporal resolution. This greatly increased the processing time without altering the final steady state; the temporal resolution given in Table 2 was sufficient for our objectives.

### 4.3 Modelling Ca^2+^ electrodiffusion with the Nernst–Plank equation

**FIGURE 5:**
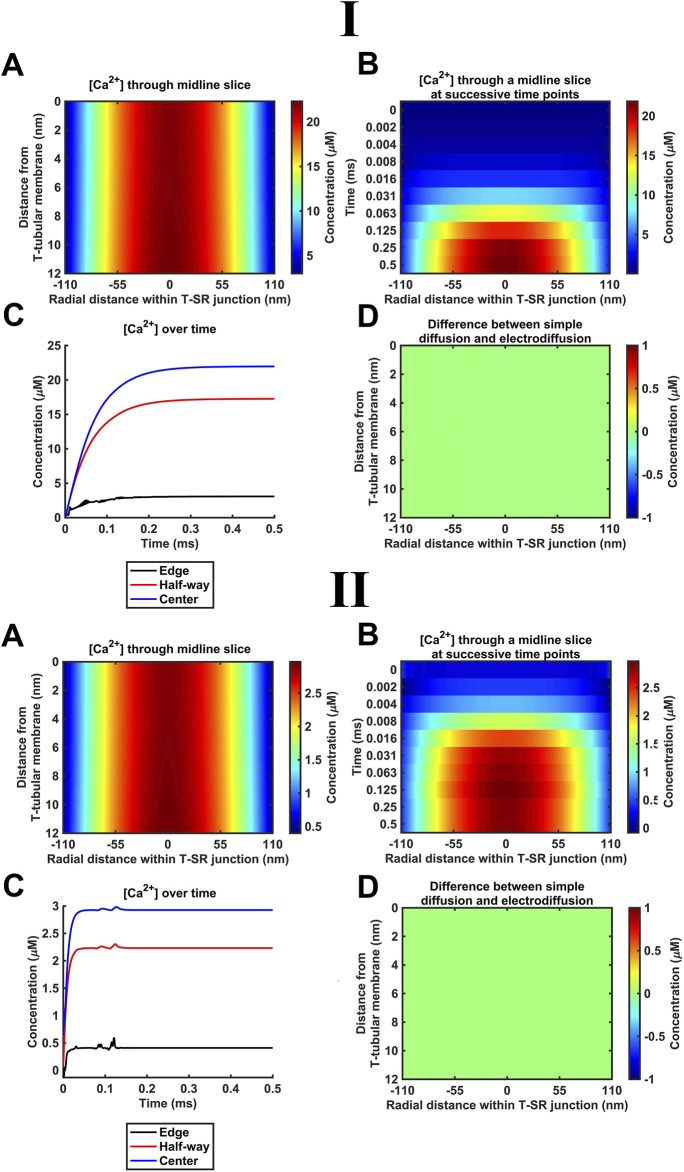
Introducing Ca^2+^ electrodiffusion to the model of the T-SR junction. (A) T-SR junctional [Ca^2+^] profile in the steady state (after 0.5 ms). (B,C) Time-dependent changes in [Ca^2+^]. (D) Difference between the heatmap in Figure 4A and the heatmap in (A) here. Note that the uniform green colour indicates no detectable difference in [Ca^2+^] after the incorporation of full electrodiffusion terms in the model. Studies made at Ca^2+^ diffusion coefficients at low and high limits of *D*_Ca_ = 4 × 10^7^ nm^2^ s^−1^ **(I)** and 7 × 10^8^ nm^2^ s^−1^ **(II)**, respectively.

The succeeding modelling stages considered contributions of both electrical potential and concentration gradients using the Nernst–Plank equation. These began with their application (Table 3 B, Equations 42, 43) to the Ca^2+^ fluxes introduced above. Thus, the concentration gradient resulting from Ca^2+^ accumulation in the T-SR junction, in turn, generates charge gradient and voltage differences. Figure 5 shows the resulting steady-state T-SR junction [Ca^2+^] profile at 0.5 ms (A) and the time evolution of [Ca^2+^] changes, following stimulus application (B, C) obtained at the low (Figure 5I) and high *D*_Ca_ limits (Figure 5II). As in the previous case, the flattened T-SR junctional geometry (Figure 5B) prevented the formation of axial gradients from the SR to T-tubular membrane but permitted significant radial concentration and charge gradients. Nevertheless, incorporation of the electrical potential term did not notably affect either the kinetics or steady-state features of domain formation. As before, [Ca^2+^] decreased from 22 µM in the centre to 3 μM (Figure 5I) and 2.9 µM to 0.4 µM at the edge at the low and high *D*_Ca_ limits (Figure 5II), respectively. Furthermore, Figure 5D shows the difference between the heatmap in Figure 4A and the heatmap in panel A here. The uniformity indicates that there is no detectable difference in [Ca^2+^] after the addition of electrodiffusion to the model at either *D*_Ca_ investigated.

### 4.4 Introducing counterions to the T-SR junction electrodiffusive model

**FIGURE 6:**
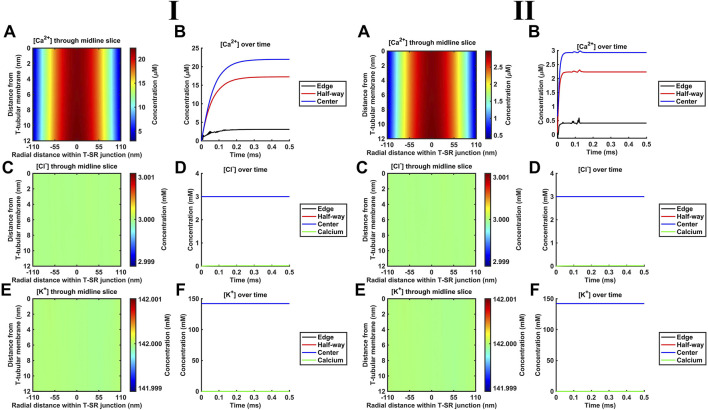
Introducing counterions (Cl^−^ and K^+^) to the electrodiffusive model. (A,C,E) Heatmaps of the respective steady-state [Ca^2+^], [Cl^−^], and [K^+^] profiles. Note similar profiles in A, as in Figures 4A, 5A, and uniform green colour in (C, E), highlighting a uniform T-SR junction [Cl^−^] and [K^+^] remaining constant and equal to resting conditions. (B) Time-dependent [Ca^2+^] changes consistent with those given in Figures 4D, 5C. (D,F) Time-dependent changes in [Cl^−^] and [K^+^] relative to [Ca^2+^]^centre^. [Cl^−^] and [K^+^] remain constant, with the counterions unaffected by the accumulation of Ca^2+^ in the junction. Changes in [Ca^2+^]^centre^ are negligible relative to [Cl^−^] and [K^+^]. Studies made at Ca^2+^ diffusion coefficients at low and high limits of *D*_Ca_ = 4 × 10^7^ nm^2^ s^−1^ **(I)** and 7 × 10^8^ nm^2^ s^−1^ **(II**), respectively.

K^+^ and Cl^−^ constitute the remaining major intracellular ions. However, they occur at mM concentrations, as opposed to the μM [Ca^2+^] concentrations considered here. Depending on the adopted low (Figure 6I) or high (Figure 6II) *D*_Ca_ limits, they would be 100- or 3-fold more diffusible than Ca^2+^. Their contributions were next included by introducing separate equations for their electrodiffusion (Table 3 C, Equations 44–47). Nevertheless, consistent with the above finding that the Ca^2+^ electrical potential term incorporated as part of the Nernst–Plank equation had little impact on Ca^2+^ distribution, neither did adding terms for K^+^ and Cl^−^, whether at the low (Figure 6I) or high (Figure 6II) *D*_Ca_ values studied. Figures 6A, C, E show steady-state heatmaps of [Ca^2+^], [Cl^−^], and [K^+^] profiles, respectively. The profiles shown in Figure 6A were similar to those of Figures 4A, 5A, suggesting unaffected [Ca^2+^] distributions.

Furthermore, Figure 6B shows time-dependent changes in [Ca^2+^] identical to those seen in Figures 5D, 6C. Furthermore, [Cl^−^] and [K^+^] were unaffected by the release of Ca^2+^ during depolarisation, remaining constant during Ca^2+^ microdomain formation. The uniformity in Figures 6C, E indicates that [Cl^−^] and [K^+^] in the T-SR junction remain constant and equal to resting conditions. In plots of the time-dependent changes in [Cl^−^] and [K^+^] relative to the Ca^2+^ concentration at the centre of the domain, [Ca^2+^]^centre^ (Figures 6D, F), [Cl^−^], and [K^+^] remained constant, accordingly unaffected by the junctional Ca^2+^ accumulation. Changes in [Ca^2+^]^centre^ were negligible relative to [Cl^−^] and [K^+^].

**FIGURE 7:**
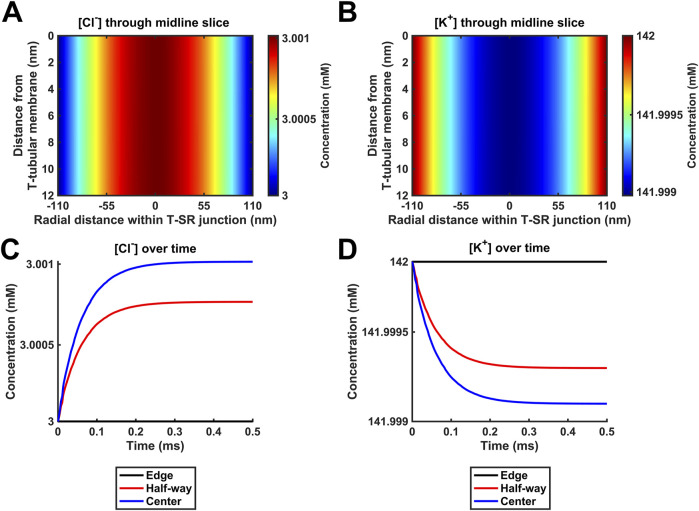
Positive control for the modelling of T-SR junction electrodiffusion. Hypothetical behaviour of Cl^−^ and K^+^ with 10^6^-fold increases in Ca^2+^ release from the SR beyond any physiologically attainable levels. **(A,B)** Heatmaps of the spatial profile of [Cl^−^] and [K^+^], respectively. **(C,D)** Time-dependent changes in [Cl^−^] and [K^+^]. Note that in this situation, [Cl^−^] and [K^+^] profiles are affected by the accumulation of a divalent cation in the T-SR junction and the subsequent charge gradient generated. The negatively charged Cl^−^ is concentrated by the accumulation of positive Ca^2+^ in the centre of the T-SR junction, while the positively charged K^+^ is repelled. Study made at Ca^2+^ diffusion coefficients at the low limit of *D*_Ca_ = 4 × 10^7^ nm^2^ s^−1^.

These control findings suggest that the magnitude of the *in vivo* T-SR junction µM [Ca^2+^] gradients did not have major physical effects on the concentrations of either Ca^2+^ or the remaining electrolyte, present at mM concentrations, whether at the low or high *D*_Ca_ limits. This has implications for the relative contributions of concentration and electrical terms on the solved Nernst–Plank equations. This notion was tested in simulations exploring the hypothetical effect of increasing Ca^2+^ flux through the SR membrane by 10^6^-fold at the low *D*_Ca_ limit (Figure 7). [Cl^−^] and [K^+^] profiles were now affected by the accumulation of a divalent cation in the T-SR junction and the subsequent charge gradient generated. Figures 7A, B show heatmaps for the steady-state spatial profiles of [Cl^−^] and [K^+^]. Figures 7C, D show the corresponding time-dependent [Cl^−^] and [K^+^] changes. They suggest that the negatively charged Cl^−^ is concentrated by the accumulation of positive Ca^2+^ in the centre of the T-SR junction, while the positively charged K^+^ was repelled.

The extensions of the original diffusion analysis of T-SR Ca^2+^ fluxes and concentrations following voltage induced SR Ca^2+^ release then proceeded from this Nernst–Planck analysis. It successively added to the analysis major intracellular electrolytes, Ca^2+^ buffering, bulk charge differences, and membrane surface-charge properties. Together, these resulted in a more physiologically realistic study of the resulting Ca^2+^ microdomains.

### 4.5 Modelling Ca^2+^ buffering

In the cytoplasm of skeletal myocytes, the major classes of Ca^2+^-binding proteins are Ca^2+^ buffers. Of these, comparisons of the major buffers CaM, troponin, parvalbumin, and myosin suggested that CaM and troponin were the most important in buffering large rapid changes in [Ca^2+^] (Robertson et al., 1981). However, troponin is restricted to the myofilaments, while CaM is mobile throughout the cell. Furthermore, CaM is the most important mobile buffer in the cytosol of skeletal myocytes (Pertille et al., 2010). It is also the major transducer of Ca^2+^ signals. It acts directly by modulating the activity of target molecules such as the RyR (McCarthy et al., 2020) and Nav (Salvage et al., 2021) or indirectly by stimulating CaM kinase II (CaMKII) and triggering signalling cascades. Therefore, CaM is an excellent Ca^2+^-binding protein to model in this context as it illustrates (i) the action of Ca^2+^ buffers on microdomain formation and (ii) the impact of these resulting microdomains on downstream Ca^2+^ signalling. Table 4 summarises the values of the main parameters used in CaM modelling.

**TABLE 4:** Summary of parameters used to model effects of calmodulin buffering.

Definition	Symbol	Value (physiological unit)	Dimensions (physiological unit)	Reference
(i) Binding reaction				
Intracellular CaM concentration	[CaM]_rest_	6	µM	Wu and Bers (2007)
CaM affinity for Ca^2+^	*K*_d_	1.5 Further values tested at *K*_d_ = 0.5 and 5 µM (published range: 0.5–5 µM)	µM	Chin and Means (2000)
Number of binding sites per CaM molecule		4		
Rate constant of Ca^2+^-CaM binding	*k*_on_	3 × 10^10^	M^−1^.s^−1^	Faas et al. (2011)
(ii) CaM diffusion				
CaM diffusion coefficient	*D*_CaM_	1.1 × 10^7^	nm^2^/s	Sanabria et al. (2008)

**FIGURE 8:**
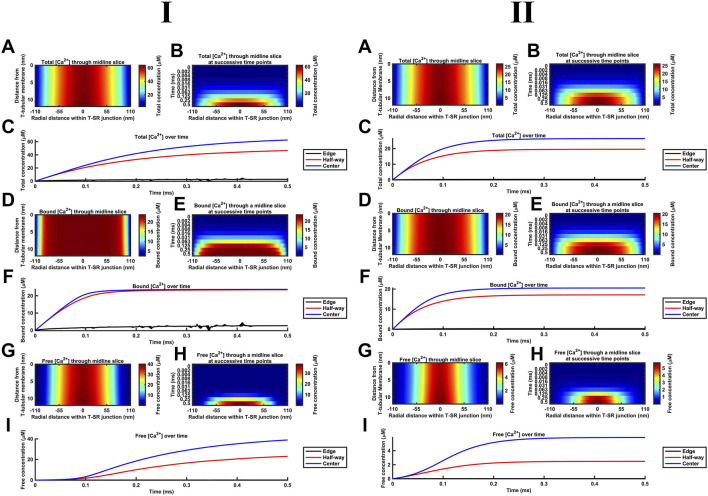
Ca^2+^ buffering by CaM increases [Ca^2+^] and [Ca^2+^]_free_ and modifies microdomain kinetics. Studies made at Ca^2+^ diffusion coefficients at low and high limits of *D*_Ca_ = 4 × 10^7^ nm^2^ s^−1^ **(I)** and 7 × 10^8^ nm^2^ s^−1^ **(II)**, respectively. (A–C) Spatial (A,B) and temporal [Ca^2+^] profiles (B,C). Profile in (A) captured at 0.5 ms. Note larger [Ca^2+^] increases than those in previously modelled situations without Ca^2+^ buffers, with maximal [Ca^2+^] in (I) reaching 52 µM (A) and slowed kinetics of microdomain formation (B,C). (D–F) Spatial (D,E) and temporal [Ca^2+^]_bound_ profiles (E,F). Profile in (D) captured at 0.5 ms. Note the saturation of CaM throughout most of the T-SR junction, with [Ca^2+^]_bound_ reaching a maximum of 24 µM (D) but a sharp decrease in [Ca^2+^]_bound_ at the edges, where [Ca^2+^]_bound_ does not exceed 3 µM (E). (B) There is a rapid [Ca^2+^]_bound_ increase to saturation within 0.1 ms, with the domain almost fully formed by 0.125 ms (F). (G–I) Spatial (G,H) and temporal [Ca^2+^]_free_ profiles **(I)**. Profile in **(G)** captured at 0.5 ms. Note the differing [Ca^2+^]_free_ compared to the [Ca^2+^]_total_ profile with a sharper decrease in [Ca^2+^]_free_ from the centre to the edge of the junction as Ca^2+^ gets heavily buffered once it decreases below the threshold for CaM saturation. There is a slower [Ca^2+^]_free_ increase than either [Ca^2+^]_bound_ or [Ca^2+^] (H,I) and sigmoid time course of [Ca^2+^]_free_ with an initial 0.1-ms lag-phase attributable to Ca^2+^ buffering by CaM in the centre of the T-SR junction **(I)**.

Figure 8 summarises the impact of CaM on T-SR junctional [Ca^2+^], following the activation of SR Ca^2+^ release at the low (Figure 8I) and high *D*_Ca_ limits (Figure 8II). The modelling (Table 3 D, Equations 47–51) used the Nernst–Planck equation with no counterions other than the Donnan protein as the previous analyses showed that these only negligibly impacted Ca^2+^ microdomain formation but greatly increased the complexity and computational load entailed by the model. Our model output the free Ca^2+^ concentration, [Ca^2+^]_free_, allowing comparisons with the computations in above and previous studies (Bardsley et al., 2021), the concentration of Ca^2+^ bound to CaM, [Ca^2+^]_bound_, relevant to its physiological regulatory properties, and the total of these concentrations, [Ca^2+^] = [Ca^2+^]_total_, reflecting the effectiveness of Ca^2+^ microdomain formation. All these parameters reflect the end result of modelling the T-SR geometry, ion-diffusion coefficients, and Ca^2+^-CaM-binding properties, following activation of SR Ca^2+^ release into the T-SR space.

First, in the presence of buffer, the spatial (Figures 8A, B) and temporal [Ca^2+^] profiles (Figure 8C) demonstrated larger eventual increases in [Ca^2+^] than in modelling situations without Ca^2+^ buffers, reaching differing maximal [Ca^2+^] of 60 and 30 μM at the low and high *D*_Ca_ limits, respectively (Figure 8A). The corresponding spatial (Figures 8D, E) and temporal [Ca^2+^]_bound_ profiles (Figure 8F) demonstrate that CaM was saturated throughout most of the T-SR junction, with [Ca^2+^]_bound_ reaching more similar maxima of 24 and 20 μM at the low and high *D*_Ca_ limits, respectively (Figure 8D). Nevertheless, the concentration decreased sharply at the edges to extents more marked in the low *D*_Ca_ limit, whence [Ca^2+^]_bound_ did not exceed 3 μM at the edges of the domain (Figure 8E). Finally, the spatial (Figures 8G, H) and temporal [Ca^2+^]_free_ profiles (Figure 8I) demonstrate spatial [Ca^2+^]_free_ profiles after 0.5 ms, differing from that of [Ca^2+^] at both the low and high *D*_Ca_ limits. Thus, there was a sharper decrease in [Ca^2+^]_free_ from the centre to the edge of the junction, suggesting that Ca^2+^ gets heavily buffered once it decreases below a threshold for CaM saturation. However, in the present system in which there is a steady-state Ca^2+^ flux through as opposed to an equilibrium quantity of Ca^2+^ within the T-SR junction, [Ca^2+^]_free_ was not reduced but increased relative to results obtained in the absence of a buffer. However, low and high *D*_Ca_ cases yielded different maximal [Ca^2+^]_free_ of 40 and 6 µM. Thus, increasing *D*_Ca_ decreased maximal [Ca^2+^], but this was mainly accounted for by reductions in maximal [Ca^2+^]_free_, and there was relatively little change in [Ca^2+^]_bound_. [Ca^2+^]_free_ was maximal in the centre but sharply decreased in the outer half of the junction. Comparing Figures 8A, G revealed that the [Ca^2+^]_free_ microdomain was highly spatially restricted and almost entirely confined to the inner half of the T-SR junction.

In general, the high [Ca^2+^] in the centre of the T-SR junction thus overcame the CaM buffering capacity, while the lower [Ca^2+^] towards the edge was heavily buffered. As such, during microdomain formation, Ca^2+^ accumulation may transiently and locally exceed buffering capacity, but this does not spread and remains a highly localised phenomenon. Indeed, although CaM was rapidly saturated at the centre of the domain, [Ca^2+^]_bound_ was low at the edge of the T-SR junction, indicating that a significant buffering capacity remained for CaM.

Second, the presence of a molecule binding and slowing down Ca^2+^ diffusion also altered the kinetics of microdomain formation. The kinetic portrayals (Figures 8B, C) show a significant slowdown in the kinetics of [Ca^2+^] microdomain formation. This was more marked with the low *D*_Ca_ values. In the latter case, there was still a significant evolution of the microdomain between 0.25 ms and 0.5 ms (Figure 8B). The [Ca^2+^] plot against time did not attain a plateau in the interval studied, indicating that steady state was not reached even by 0.5 ms. With the high D_Ca_ values, [Ca^2+^] increased more rapidly, attaining a plateau by 2 ms. In contrast, Figures 8E, F show that [Ca^2+^]_bound_ increases very rapidly, reaching saturation within 0.1 ms, and with the domain almost fully formed by 0.125 ms with both the low and high *D*_Ca_ values (Figure 8F). However, Figures 8H, I demonstrate that [Ca^2+^]_free_ increases more slowly than both [Ca^2+^]_bound_ and [Ca^2+^], an effect more marked at low *D*_Ca_. In the latter situation, plots of [Ca^2+^]_free_ in the centre of the T-SR junction followed a sigmoid evolution, with an initial 0.1-ms lag phase attributable to Ca^2+^ buffering by CaM (Figure 8I). [Ca^2+^]_free_ kinetics were considerably more rapid at the high *D*_Ca_ value, reaching their maximum values by 0.2 ms, following the onset of the Ca^2+^ influx while remaining more rapid than the corresponding [Ca^2+^] and [Ca^2+^]_bound._

Third, during the early stages of Ca^2+^ release, most of the Ca^2+^ was bound by CaM, with little change in [Ca^2+^]_free_, while CaM was rapidly saturated at both tested *D*_Ca_. This shows potential physiological significance: saturated CaM-Ca^2+^ and free Ca^2+^ have distinct signalling properties—CaM regulates downstream effectors (e.g., CaMKII, RyR1, Nav, and SK), while free Ca^2+^ modulates other proteins [e.g., RyR1, Nav, BK, and NCX (see *Discussion*)] or interacts with voltage-sensitive proteins by means of its charge. Figure 8 also highlights these two important regulators of the T-SR junction evolving with distinct temporal kinetics during an AP: where these two pathways converge (e.g., regulation of RyR1), the actions of CaM and free Ca^2+^ are staggered due to Ca^2+^ buffering.

### 4.6 Buffer-mediated Ca^2+^ trapping within the T-SR junction

**FIGURE 9:**
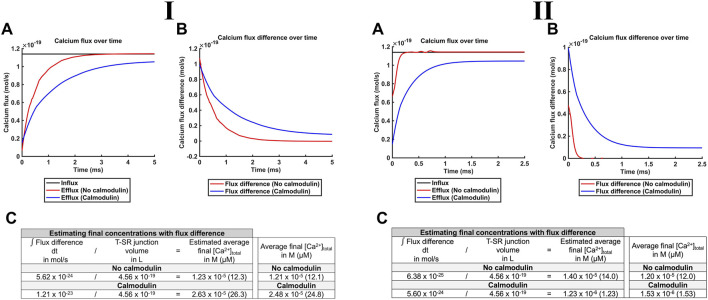
CaM traps Ca^2+^ in the centre of the T-SR junction, enhancing microdomain formation. Studies done at Ca^2+^ diffusion coefficients at low and high limits of *D*_Ca_ = 4 × 10^7^ nm^2^ s^−1^ **(I)** and 7 × 10^8^ nm^2^ s^−1^ **(II)**, respectively. (A) Magnitude of Ca^2+^ fluxes into, through face F2, the SR membrane and out of the modelled T-SR junction through face F3, opening onto the bulk cytosol. Ca^2+^ influx is the same in the presence and absence of CaM, but Ca^2+^ efflux is greatly reduced in the presence of CaM, leading to a greater (B) flux difference obtained by subtracting the Ca^2+^ efflux from the influx. (C) Results of the control exploration exemplifying different levels of the consequent Ca^2+^ trapping before and following the introduction of CaM, confirming conservation between the average final [Ca^2+^], estimated from the flux difference and T-SR junction volume, and the actual final concentration, obtained from averaging the [Ca^2+^] at the end of the simulation.

Of potential physiological implications of these features, first, noteworthy were the higher levels of [Ca^2+^]_free_ attained in the presence of CaM than those in its absence. This is compatible with buffering by CaM reducing the leakage of [Ca^2+^]_free_ into the bulk cytosol. Ca^2+^-CaM binding then effectively traps Ca^2+^ in the T-SR junction, causing the [Ca^2+^]_free_ microdomain to become spatially restricted (Figure 8G). Such T-SR junctional Ca^2+^ trapping could be directly modelled by analysing the magnitude of Ca^2+^ fluxes into and out of the junction in the presence and absence of CaM, at both the low and the high *D*_Ca_ values (Figures 9I, II, respectively; Table 3 E, Equations 52–54). The Ca^2+^ influxes through the SR membrane face F2 and effluxes through the T-SR junctional edge face F3, opening onto the bulk cytosol.

Figure 9A plots the modelled magnitudes of T-SR junction Ca^2+^ influxes and effluxes. The influx of Ca^2+^, $\Phi_{\text{influx}}$ = 1.14 × 10^−19^ mol/s for the modelled *J*_influx_ = 3.00 × 10^−24^ mol/(nm^2^ s), was constant through time whether CaM was present or absent. However, the Ca^2+^ efflux $\Phi_{\text{efflux}}$ was greatly reduced in the presence of CaM. This led to a greater flux difference obtained by subtracting Ca^2+^ efflux from the influx, yielding the net Ca^2+^ flux into the T-SR junction (Figure 9B), $\Phi_{\text{influx}}-\Phi_{\text{efflux}}$ . Hence, the presence of CaM enhances the trapping of Ca^2+^ within the junction, further restricting the microdomain, explaining the high [Ca^2+^]_free_ reached. Its integration over time developed in *Theory* could provide the mean [Ca^2+^] accumulated at the end of any given test interval (0, τ ), <[Ca^2+^]>_τ_ (Figure 9C, column 1), for a T-SR junction of volume $\left(\frac{\pi wd^{2}}{4}\right)$ (Figure 9C, column 2). The presence of CaM thus increases <[Ca^2+^]>_τ_ (Figure 9C, column 3). This result could be confirmed by a comparison with the corresponding <[Ca^2+^]>_τ_ value derived from the *spatial* integral of the actual final concentrations through the cross-sectional area of the T-SR junction: (Figure 9C, column 4). Thus, the final <[Ca^2+^]>_τ_. estimated over time using flux difference matched the actual final concentrations over space (Table 3 E, Equations 52–54; Figure 9C, compare columns 3 and 4), with a slight difference attributable to the stiffness of the Neumann BC, leading to noisy concentrations and fluxes (e.g., Figure 8F). This confirmation of overall Ca^2+^ conservation was a useful validation of the modelling process (Figures 9B, C).

### 4.7 Impact of variations in CaM affinity on [Ca^2+^] and microdomain kinetics

The exact CaM affinity for Ca^2+^, its *K*_d_ (Table 3 D, Equation 50), is difficult to measure with different values quoted in the literature, varying with the measurement method (surface plasmon resonance and radioisotope displacement), conditions (*in vitro*, *in vivo*, and ion concentrations), what is measured (EF-hand affinity and apparent affinity), the specific CaM (different isoforms and species), and the conformation (R-state and T-state) (Faas et al., 2011). Independent of the precise conditions, *K*_d_ varies with conformational changes and post-translational modifications. A T- to R-state conformational change increases the affinity 100-fold (Faas et al., 2011). Ser^101^ phosphorylation by casein kinase II significantly increases the affinity. Ser^101^ is near the third EF-hand (Ca^2+^-binding motif); the negative charge on the phosphate group enhances cation binding (Aiuchi et al., 1991). These inconsistencies complicate the choice of a specific value, but consensus values for *K*_d_ fall between 0.5 µM and 5 µM (Chin and Means, 2000). We used our model to explore the consequences of such variations in Ca^2+^ microdomain formation and CaM signalling.

**FIGURE 10:**
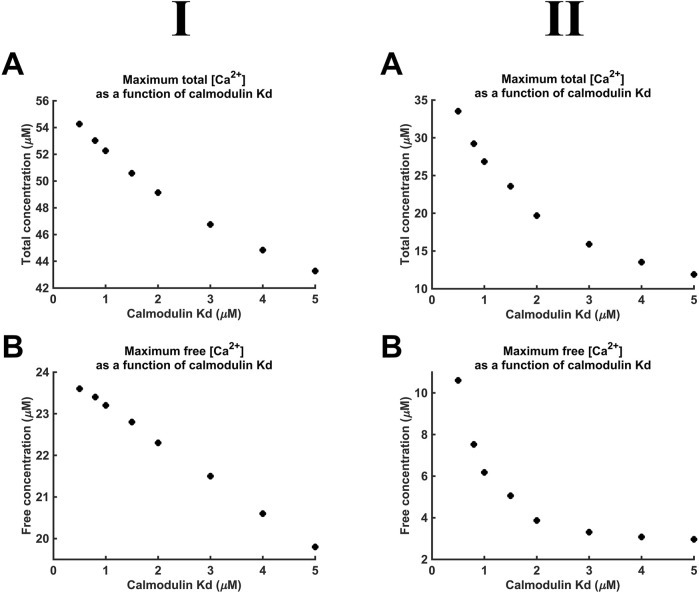
[Ca^2+^] and [Ca^2+^]_free_ as functions of CaM Ca^2+^ binding constant *K*_d_. Studies done at Ca^2+^ diffusion coefficients at low and high limits of *D*_Ca_ = 4 × 10^7^ nm^2^ s^−1^ **(I)** and 7 × 10^8^ nm^2^ s^−1^ **(II)**, respectively. Maximal microdomain [Ca^2+^] (A) and [Ca^2+^]_free_ (B) with varying CaM Ca^2+^ affinities within the reported physiological range (0.5 µM–5 µM). [Ca^2+^] decreases as the affinity of CaM decreases (higher *K*_d_).

Figure 10 demonstrates decreases in maximal [Ca^2+^] and [Ca^2+^]_free_ with reductions in CaM affinity at both *D*_Ca2+_ values tested. At lower *D*_Ca2+_, [Ca^2+^] notably decreased from 55 to 43 μM, and [Ca^2+^]_free_ showed a smaller, 24 µM to 20 μM, decrease. At higher *D*_Ca2+_, [Ca^2+^] showed a greater proportional decrease from 35 to 11 μM, and [Ca^2+^]_free_ showed a decrease from 10 µM to 3 μM. The smaller decrease of [Ca^2+^]_free_ compared to that of [Ca^2+^] is attributable to the reduced CaM saturation at lower affinity. Thus, at *K*_d_ = 0.5 µM and 5 μM, 98.3% and only 82.5% respectively of available CaM Ca^2+^-binding sites were occupied. The linear relationships between *K*_d_, [Ca^2+^], and [Ca^2+^]_free_ were shallow.

**FIGURE 11:**
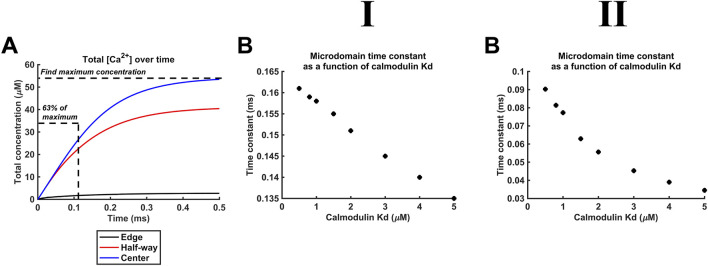
Microdomain formation slows with increased CaM affinity for Ca^2+^. Studies done at Ca^2+^ diffusion coefficients at low and high limits of *D*_Ca_ = 4 × 10^7^ nm^2^ s^−1^ **(I)** and 7 × 10^8^ nm^2^ s^−1^ **(II)**, respectively. (A) Calculation of the time constant of microdomain formation in hypothetical [Ca^2+^] plots against time. (B) Dependence of the microdomain time constant on CaM *K*_d_.

Additional to maximal [Ca^2+^], *K*_d_ variations also affect the kinetics of microdomain formation. The rate of microdomain formation was represented by a time constant given by the length of time necessary to reach 63% (1–1/*e*) of maximal [Ca^2+^] (Figure 11A). The high (Figure 11II), as opposed to the low *D*_Ca2+_ (Figure 11I), value was associated with lower time constants. The time constants decreased with decreasing CaM affinity for Ca^2+^, i.e., microdomains form faster at lower CaM affinity and higher *D*_Ca2+_ (Figure 11B). However, a 10-fold reduction in affinity leads only to a 15% reduction in the time constant. The kinetics of microdomain formation are thus relatively robust to altered CaM affinity.

**FIGURE 12:**
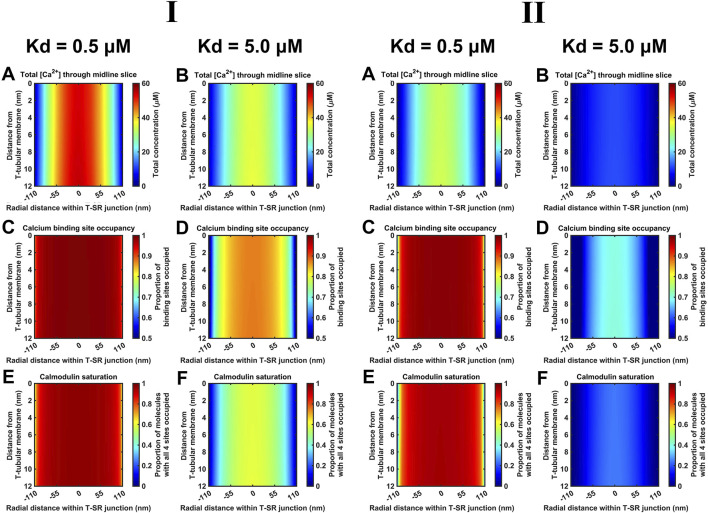
Ca^2+^-binding sites and CaM saturation as functions of the CaM affinity for Ca^2+^. Studies done at Ca^2+^ diffusion coefficients at low and high limits of *D*_Ca_ = 4 × 10^7^ nm^2^ s^−1^ **(I)** and 7 × 10^8^ nm^2^ s^−1^ **(II)**, respectively. T-SR junctional profiles of [Ca^2+^] (A,B), proportion of occupied CaM-Ca^2+^-binding sites (C,D), and proportion of CaM molecules saturated with all four Ca^2+^-binding sites occupied (E,F) for high (A,C,E) and low CaM affinity (B,D,F), respectively. Note ∼30% maximum [Ca^2+^] variations from 55 μM at high to 40 μM at low affinity in (I), but sharp spatial decreases towards the T-SR junction edges (A,B). Virtually, all the Ca^2+^-binding sites occupied by Ca^2+^ at high affinity and only a fraction of the binding sites are occupied at the edges of the T-SR junction at low affinity (C,D). Virtually, all the CaM molecules fully saturated with all four Ca^2+^-binding sites were occupied at high affinity, whereas ∼50% of the CaM molecules saturated at low affinity in the centre of the domain were occupied at low affinity; these proportions decrease sharply towards the edges of the T-SR junction.

Finally, *K*_d_ variations steeply altered CaM-Ca^2+^-binding site occupancies through the microdomain. Figure 12 shows eventual [Ca^2+^] (A, B), Ca^2+^-binding site occupancies (C, D) and CaM saturation (E, F) profiles at high (*K*_d_ = 0.5 µM) (A, C, E) and low (*K*_d_ = 5 µM) CaM affinities (B, D, F) at both low (Figure 12I) and high *D*_Ca2+_ values tested (Figure 12II). [Ca^2+^] variations were relatively small: maximum [Ca^2+^] decreased by ∼30% from 55 μM at high to 40 μM at low affinity at low *D*_Ca2+_ and from 35 μM to 10 μM at high *D*_Ca_. At both *D*_Ca2+_ values, there were sharp spatial decreases towards the T-SR junction edges. In contrast, 93% of all CaM molecules were saturated at high affinity, decreasing to 47% saturation at low affinity at low *D*_Ca2+_. Similarly, 90% of CaM molecules were saturated at high affinity, decreasing to 25% saturation at low affinity, at low *D*_Ca2+_. However, at the T-SR junction edges, virtually all and only a fraction of Ca^2+^-binding sites were occupied whether at high or low CaM affinity, respectively, at both tested *D*_Ca2+_ (Figures 12C, D). Correspondingly, in the domain centre, virtually all and only ∼50% of the CaM molecules were saturated with all four Ca^2+^-binding sites occupied. These proportions decreased sharply towards the edges of the T-SR junction (Figures 12E, F). Thus, variations in CaM affinity following conformational and post-translational modifications, in addition to [Ca^2+^] variations, could modify T-SR junction Ca^2+^ microdomains. This could affect CaM-mediated Ca^2+^ signal transduction. Such actions would be superimposed on CaM’s intrinsic regulatory properties: CaM has four Ca^2+^-binding sites, all of which require occupation to activate CaM’s regulatory role whether on RyR or CaMKII.

### 4.8 Action of accumulated T-SR junction Ca^2+^ on membrane potentials

**FIGURE 13:**
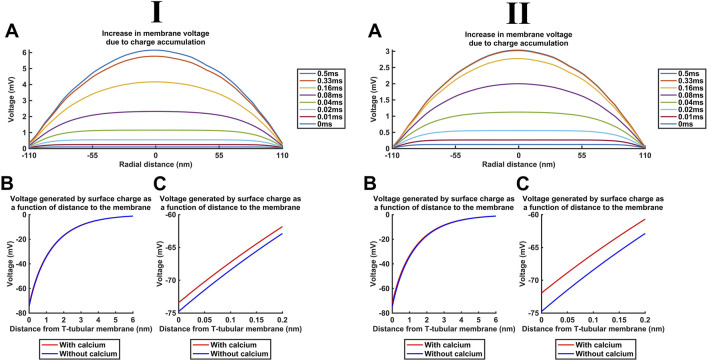
Ca^2+^ release into the T-SR junction alters the bulk cytosolic and Stern layer potentials. Studies done at Ca^2+^ diffusion coefficients at low and high limits of *D*_Ca_ = 4 × 10^7^ nm^2^ s^−1^ **(I)** and 7 × 10^8^ nm^2^ s^−1^ **(II)**, respectively. (A) Increase in *V*_i_ resulting from the accumulation of positive charges in the T-SR junction cytosol, calculated with the charge difference approach. Even at the centre with the greatest accumulated Ca^2+^, the total [Ca^2+^] only causes a +6-mV increase in *V*_*i*_, at the end of the simulation (0.5 ms). (B) Stern layer potential *V*_*σ*i_(*x*) as a function of distance, *x* (see Figure 3). The negative charge of phospholipids, σ _i_, generates −75 mV of surface (*x* = 0) potential, decaying to 0 mV within 6 nm. Zoom-in (C) of the first *x* < 0.2 nm, showing that the predicted [Ca^2+^]_free_ changes at the centre of the domain and, at the end of the simulation (t = 0.5 ms), only causes a +1.4-mV surface potential increase.

Additional to the above cytosolic actions of kinetically distinct free and CaM-bound Ca^2+^ changes on important T-SR junctional molecules including Nav1.4 and RyR1, the released Ca^2+^ potentially affects T-tubular, Δ*V*, and SR transmembrane potentials. These could also affect membrane protein function. We explored these effects for the high concentrations [Ca^2+^] = 52 µM and [Ca^2+^]_free_ = 28 µM predicted here for low *D*_Ca2+_ (Figure 13I) and [Ca^2+^] = 30 µM and [Ca^2+^]_free_ = 6 µM for high *D*_Ca2+_ (Figure 13II). These yielded similar results, which are described for low *D*_Ca2+_

First, Figure 13A shows the increase in the bulk intracellular membrane potential *V*_i_ calculated with the charge difference approach (Table 3 F, Equations 55, 56). Even the highest [Ca^2+^] = 52 μM at the T-SR junction centre at radial distance = 0 nm only generated a +6-mV increase in *V*_i_ (Figure 13A) and, therefore, ∆*V*.

Second, in the absence of Ca^2+^, both inner- and the outer-membrane leaflets possess a surface charge of densities σ _I_ and σ _o_, respectively. σ _i_ measurements vary with the cell line and membrane composition, possibly reflecting differing proportions of 1,2-dipalmitoyl-sn-glycero-3-phosphocholine (DPPC), 1,2-dipalmitoyl-sn-glycero-3-phosphoglycerate (DPPG), and 1,2-dioleoyl-sn-glycero-3-phosphocholine (DOPC), between −5 μC/cm^2^ and −10 μC/cm^2^, with a consensus value of approximately −5 μC/cm^2^ (Fuhs et al., 2018; Olivotto et al., 1996; Ouyang et al., 2021). The resulting Stern-layer potential *V*_σi_(*x*) decays from −75 mV at the membrane surface at distance *x* = 0 nm to 0 mV at *x* = 6 nm (Figures 13B, C). Ca^2+^ adsorption on the surface of the plasma membrane “screens” some of this negative surface charge, reducing σ _i_. The reported values of the affinity of such Ca^2+^-phospholipid binding vary with *K*_d_ between 1 mM and 150 mM (Bers et al., 1985; Deplazes et al., 2021; McLaughlin et al., 1981; Melcrová et al., 2016). The modelling here (Table 3G, Equation 57) adopted *K*_d_ = 10 mM within this range.

Table 5 summarises the parameter values used in surface potential modelling altering such surface charges. A maximum [Ca^2+^]_free_ at the centre of the domain, at the end of the simulation (*t* = 0.5 ms) reaching 28 µM, as modelled here, generated a +1.4-mV surface potential increase at *x* = 0 nm. This change is not matched at the outer leaflet, effectively resulting in only a small, +1.4-mV increase in ∆*V* (Figure 13D). The outer-membrane leaflet generates similar potentials, but small variations in adsorption at extracellular mM Ca^2+^ concentrations would only minimally affect the surface potential at the outer-membrane surface.

**TABLE 5:** Summary of parameters used to calculate membrane potential changes.

Definition	Symbol	Value (physiological unit)	Dimensions (physiological unit)	Reference
(i) Charge difference modelling				
Plasma membrane-specific capacitance	*C*_m_	1 × 10^−20^	F/nm^2^	Satoh et al. (1996)
(ii) Surface charge modelling				
Plasma membrane inner-leaflet surface-charge density	σ_i_	−5	µC/cm^2^	Fuhs et al. (2018), Olivotto et al. (1996), and Ouyang et al. (2021)
*K*_d_’ of Ca^2+^ binding to membrane phospholipids	*K*_d_’	10	mM	Bers et al. (1985), Deplazes et al. (2021), McLaughlin et al. (1981), and Melcrová et al. (2016)
Debye length in the cytosol	$\kappa^{-1}$	1.5	nm	Cossins et al. (2011), Khunpetch et al. (2022), and Wennerström et al. (2020)

These changes in ∆*V* are additive, summing to a maximum change of 7.4 mV in ∆V. This is small in relation to either resting membrane potentials of ∼−90 mV or the action potential-mediated excursion to +40 mV in skeletal myocytes (Filatov et al., 2005). These findings confine Ca^2+^ release to a modification of voltage-dependent ion channel activation and inactivation, as opposed to the transmembrane field.

## 5 Discussion

Ca^2+^ microdomains can form at the mouths of Ca^2+^ channels during cell signalling due to large transmembrane Ca^2+^ release/entry gradients and relatively poor Ca^2+^ cytosolic diffusibility. They are likely accentuated in cellular structures such as at skeletal muscle T-SR triad junctions, where closely apposed membranes further restrict Ca^2+^ diffusion. Such T-SR junctions are strategic in excitation–contraction coupling (Franzini-Armstrong and Nunzi, 1983; Franzini-Armstrong et al., 1999; Kelly, 1969). Here, Cav1.1-DHPR1 conformational changes allosterically activate directly coupled SR-RyR1-Ca^2+^ channels (Huang et al., 2011) and possibly other adjacent, coupled SR-RyRs (Huang, 2001; Marx et al., 1998), initiating intracellular SR-Ca^2+^ release. In contrast, in cardiac muscle dyad junctional sites, Cav1.2-mediated Ca^2+^ entry initiates a Ca^2+^-induced, RyR2-mediated SR-Ca^2+^ release (Cannell and Soeller, 1997; Endo, 2009; Fabiato and Fabiato, 1975; Soeller and Cannell, 1997). This could potentially yield contrasting, more nonlinear effects on junctional Ca^2+^ levels that could merit future detailed study.

However, their small size and difficulties inherent in their direct experimental study leave the physiological events within Ca^2+^ microdomains poorly understood. Nevertheless, modelling studies may provide useful insights into processes within this space. Recent modelling of simple Ca^2+^ diffusion suggested that such microdomains could transiently and locally reach concentrations 1,000-fold greater than the remaining bulk resting cytosolic [Ca^2+^] (Bardsley et al., 2021). The present study more realistically incorporated effects of additional *in vivo* factors, including charge gradients, counterions, and Ca^2+^ and osmotic buffers, on the development and properties of such Ca^2+^ microdomains. It assessed their possible contributions to cytosolic and surface membrane signalling. Adding to the previous report, it explored effects of voltage gradients consequent upon Ca^2+^ accumulation and additional electrodiffusive effects of the *in vivo* counterions K^+^ and Cl^−^ and osmotic-buffering anions. It similarly assessed the effect of varying 10^7^∼10^8^ nm^2^ s^−1^ reported Ca^2+^ diffusion values, further bearing in mind restricted T-SR space conditions potentially not representative of the whole-cell cytoplasm. Adopting the *D*_Ca2+_ value proposed by Baylor and Hollingworth (1998), *D*_Ca2+_ = 7 × 10^8^ nm^2^ s^−1^, a two-fold smaller *D*_Ca2+_ than the free value assumed a myoplasmic viscosity 2-fold than that of a simple salt solution (Kushmerick and Podolsky, 1969). It excluded effects of Ca^2+^-sequestering membrane-bound organelles, likely true within the T-SR space, and of cytoplasmic Ca^2+^ buffers. However, even the T-SR-restricted space likely includes Ca^2+^ buffers additional to CaM, affecting *D*_Ca2+_. Including the entire bulk cytoplasmic Ca^2+^-binding capacity would have yielded a 50-fold *D*_Ca2+_ reduction to 2.8 × 10^7^ nm^2^ s^−1^ (Kushmerick and Podolsky, 1969). Nevertheless, the adopted *D*_Ca2+_ value could provide an upper computational limit. This could be compared with the results from a lower *D*_Ca_ limit of 4.0 × 10^7^ nm^2^ s^−1^ based on empirical isotope and mini-electrode measurements (al-Baldawi and Abercrombie, 1995). This would comprehensively cover variations in the results arising from reported *D*_Ca2+_ variations. Furthermore, with these low and high D_Ca2+_ limits, the modelling then incorporated Ca^2+^ buffering and its actions upon both free [Ca^2+^]_free_ and total [Ca^2+^], which could impact cytosolic Ca^2+^ signalling. It also considered consequences for the T-tubular membrane potential that might also directly impact voltage-sensitive proteins. Finally, comparing the results at the low and high *D*_Ca2+_ limits lent security to the inferences here as applicable through the entire range of reported *D*_Ca2+_. They also yielded further insights into the relative effects of *D*_Ca2+_ and CaM buffering on domain characteristics.

Findings from such an analysis have implications in skeletal muscle physiology, detailed and referenced below, for (1) the possible importance of CaM regulating the kinetics and the extent of Ca^2+^ microdomain formation and its own role in local T-SR junctional Ca^2+^ signalling. It exerted relatively (2) minor effects upon T-tubular and possibly SR transmembrane potentials. Nevertheless, both Ca^2+^ and CaM likely (3) activate multiple RyR regulatory sites. They may also exert (4) inhibitory actions in skeletal (Na_v_1.4) and cardiac muscle Na^+^ channel (Nav1.5) C-terminal EF-like hand motifs and isoleucine–glutamine (IQ) domain regions or binding sites between Na_v_ domains III and IV, (5) activating actions on both “big” (BK)- and small-conductance (SK1, SK2, and SK3) K^+^ channels; both (4) and (5) may reduce skeletal muscle membrane excitability. They also potentially (6) modify Na^+^/Ca^2+^ exchanger activity. They, thus, potentially contribute to important physiological and clinical translational situations.

Several, some counter-intuitive, predictions illustrating the need for such a full quantitative analysis of the properties of complex systems of this kind emerged. First, at both *D*_Ca2+_ values studied, electrodiffusion little impacted microdomain formation under the adopted *in vivo* electrolyte and osmotic conditions. Full Nernst–Plank modelling yielded similar Ca^2+^ microdomain formation properties as the simple Fick diffusion analysis, aligned to previous reports (Bardsley et al., 2021). Further introducing K^+^ and Cl^−^ counterions into the model also did not significantly alter the microdomain [Ca^2+^]. The higher *D*_Ca2+_ did reduce the attained domain [Ca^2+^]_free_ but sped up the kinetics with which this was reached. In contrast, control introductions of 10^6^ greater, unphysiological Ca^2+^ influx terms did demonstrate significant differences. These findings together indicate that the electrical potential term of the Nernst–Plank equation resulting from the charge accumulation, resulting from Ca^2+^ influx into the domain, contributed little to ion movement and to the resulting T-SR junction [Ca^2+^] under the adopted *in vivo* conditions.

Second, computations with either *D*_Ca2+_ quantitatively demonstrated for the first time that CaM is a potential major regulator of both the kinetics and extent of Ca^2+^ microdomain formation. This could indicate its major role in Ca^2+^ signalling at T-SR junction microdomains: CaM is the major mobile cytosolic buffer in skeletal myocytes. We demonstrate an unexpected result that at the microdomain level with a sustained Ca^2+^ entry, such buffers bind their target, reduce its diffusibility, and trap it in the microdomain, leading to an overall increase in free target Ca^2+^ concentration. Introducing CaM thus increased both [Ca^2+^] and [Ca^2+^]_free_. Further modelling attributed this effect to Ca^2+^ trapping by the relatively immobile CaM, restricting Ca^2+^ efflux. The [Ca^2+^] reached transiently exceeded the CaM buffering capacity, particularly at the centre of the junction for durations of 0.5 ms. Furthermore, [Ca^2+^], [Ca^2+^]_free_, and microdomain kinetics were sensitive to the CaM affinity for Ca^2+^. The latter could vary *in vivo* with post-translational modifications, its existence in its active or inactive states, and the exact cellular conditions. Thus, variations in the *K*_d_ of CaM altered the Ca^2^ efflux leaving the T-SR junction of the F3 face. Calmodulin, thus, provides a crucial read-out, modulating the function of the main effectors of the T-SR space. In these simulations performed in the presence of the CaM buffer, higher *D*_Ca2+_ resulted in a decrease in the attained [Ca^2+^] reflecting a decrease in [Ca^2+^]_free_. However, it had smaller effects on [Ca^2+^]_bound_ than did the buffering by CaM. The concentration profiles were similar at high and low *D*_Ca2+_ with sharp decreases at the domain edges. The decreases in [Ca^2+^] and [Ca^2+^]_free_ were less marked at the high *D*_Ca2+_. High *D*_Ca2+_ also reduced the extent to which buffering slowed the kinetics of microdomain formation.

Third, we explored the influences of T-SR junction Ca^2+^ domain formation on T-tubular, and possibly, SR transmembrane potentials, using both *D*_Ca2+_ values. This yielded closely concordant results, which are therefore described for the low-*D*_Ca2+_ case. The charge differences resulting from cytosolic Ca^2+^ accumulation contributed a profile of voltage change ∆*V*, which, however, did not exceed ∼6 mV. Ca^2+^ adsorption to the inner leaflet of the T-tubular membrane additionally generated a surface Stern potential, but this did not exceed 1.4 mV, matching some previous reports (Catacuzzeno et al., 2008). Other modelling had estimated that Ca^2+^ adsorption on the inner-membrane leaflet produced a +30-mV ∆*V* on elevating [Ca^2+^]_i_ to 2 µM (Pizarro et al., 1991). However, this had adopted extremely higher-than-accepted values of Ca^2+^–phospholipid affinity, close to the *K*_d_ = 20 μM of CaM, contrasting with the accepted reported mM *K*_d_’ range of phospholipid–Ca^2+^ (Bers et al., 1985; Deplazes et al., 2021; McLaughlin et al., 1981; Melcrová et al., 2016). With those latter *K*_d_’ values, ∆*V* changes predicted by Pizarro et al. (1991) would have required physiologically unrealistic, mM, changes in [Ca^2+^]_i_ here.

**FIGURE 14:**
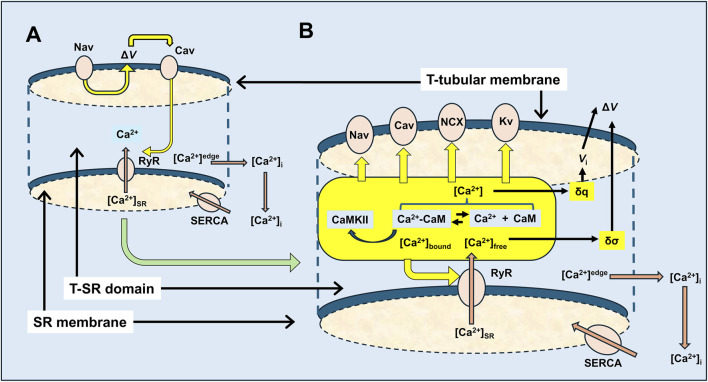
Feedforward vs. feedback actions in excitation–contraction coupling involving possible T-SR junction Ca^2+^ domains. **(A)** Classical feedforward events initiated by Nav-mediated depolarisation leading to Cav activation, triggering the RyR-mediated release of SR-stored Ca^2+^, by either direct allosteric (in skeletal muscle) or Ca^2+^ entry-induced (in cardiac muscle) Cav-RyR coupling. Released Ca^2+^ is eventually retrieved into the SR by SR-Ca^2+^-ATPase (SERCA)-mediated transport. These initial events are contrasted with **(B)** feedback events, following RyR-mediated SR-Ca^2+^ release involving a T-SR junctional Ca^2+^ microdomain space in which the released Ca^2+^ is buffered and, thus, trapped by cytosolic CaM, resulting in reactions modifying the equilibrium Ca^2+^ + CaM ↔Ca^2+^-CaM, in which Ca^2+^, CaM, and Ca^2+^-CaM are all key signalling molecules. These potentially exert feedback effects exemplified here in the T-tubular membrane molecules, Nav and Cav, that initiate excitation–contraction coupling, and in the SR membrane, Ca^2+^-releasing RyR, with potential extensions to Kv, NCX, and cellular metabolic signalling.

Our present studies adopted physiologically realistic T-SR junctional structures, participating ions and their concentrations, and Ca^2+^, particularly CaM-mediated, buffering. They predicted Ca^2+^ accumulation enhanced by the restricted T-SR junctional geometry. This was further increased by the inclusion of CaM. The resulting Ca^2+^-CaM buffering then further increased both [Ca^2+^] and [Ca^2+^]_free_. This would result in activated levels of [Ca^2+^-CaM]. It also resulted in an increased [Ca^2+^] accumulation within and decreased Ca^2+^ efflux to the remaining cytosol from the T-SR junction. The extent and features of such Ca^2+^ microdomains could be modified by both channel-mediated Ca^2+^ fluxes and longer-term variations in CaM properties affecting its *K*_d_ (Parekh, 2008). These modifications would involve both absolute and relative free Ca^2+^, Ca^2+^-CaM, and CaM levels, all established cellular signalling agents. This has implications for previous reports, adding to known T-SR junctional feedforward excitation–contraction coupling events (Figure 14A). They suggest reciprocal feedback regulatory phenomena involving the resulting Ca^2+^ microdomains (Figure 14B).

Amongst other examples (Figure 14B) (Lei et al., 2024; Li et al., 2023; Salvage et al., 2023), the latter could first involve multiple RyR regulatory sites (Lanner et al., 2010), in turn modulating Ca^2+^ flux and microdomain formation. Both Ca^2+^ and CaM likely activate RyR1. Increased [Ca^2+^] actually increases RyR1 affinity for CaM (Rodney et al., 2000). In contrast, Ca^2+^-CaM inhibits skeletal muscle RyR1-mediated SR Ca^2+^ release. These have implications for both positive and negative homoeostatic modifications of the resulting Ca^2+^ domain, with possible physiologically important consequences (Fruen et al., 2003). Ca^2+^-CaM also activates CaMKII, which acts on specific RyR1 phosphorylation sites (Meissner, 2010), increasing Ca^2+^ release (Gehlert et al., 2015). Cardiomyocyte excitation–contraction coupling involves Ca^2+^ influxes through voltage-activated dihydropyridine receptors (DHPR2 and Cav1.2) activating Ca^2+^-induced SR-Ca^2+^ release by RyR2 (Endo, 2009; Fabiato and Fabiato, 1975) at dyad junctional complexes. Here, Ca^2+^ microdomain formation would also be of direct interest.

Second, skeletal (Na_v_1.4) and cardiac muscle Na^+^ channels (Nav1.5) possess potential Ca^2+^ and CaM-binding modulatory sites (Nathan et al., 2021). Ca^2+^ might bind directly to one or more EF-like hand motifs (Yoder et al., 2019). Alternatively, Ca^2+^-CaM could bind to the IQ domain region at their C-terminal domain following initial Ca^2+^ binding to EF-hand motifs on CaM (Gardill et al., 2019; Young and Caldwell, 2005) or to a site between Na_v_ domains III and IV (Potet et al., 2009). Navs additionally contain sites phosphorylatable by Ca^2+^-CaM-regulated CaMKII (Loussouarn et al., 2016) and protein kinase C (Bendahhou et al., 1995). Elevating [Ca^2+^]_i_ to ∼2 µM by rapid Ca^2+^ photo-release or overspill from neighbouring Ca^2+^ channels reduced Na^+^ current, *I*_Na_, *in vitro* in single-cell patch-clamped Na_v_1.4-transfected HEK293 cells and skeletal muscle cell lines. These effects were abrogated by intracellular BAPTA (Ben-Johny et al., 2014), or mutations in the CaM-Ca^2+^-binding EF hands, or the Nav1.4 C-terminal IQ domain (Ben-Johny et al., 2014; Deschênes et al., 2002; Young and Caldwell, 2005).

Fluo-3-AM and fura-PE3-AM Ca^2+^ fluorescence studies demonstrated that both the RyR-Ca^2+^ release activator caffeine and SR Ca^2+^ re-uptake inhibitor cyclopiazonic acid (CPA) (Du et al., 1994; Seidler et al., 1989) increased bulk cytosolic [Ca^2+^] in rat soleus and oesophageal striated muscle (Du et al., 1994; Pagala and Taylor, 1998; Seidler et al., 1989; Sekiguchi et al., 1999). In intact loose patch-clamped native murine skeletal muscle fibre preparations (Almers et al., 1983), caffeine and Epac-mediated RyR activation, as well as the RyR inhibitor, dantrolene, correspondingly reduced and increased Na^+^ current, *I*_Na_. Dantrolene pretreatment further abrogated the *I*_Na_ inhibitory effects of caffeine or Epac activation (Matthews et al., 2019; Sarbjit-Singh et al., 2020). However, CPA paradoxically increased *I*_Na_, preserving its time course, steady-state half-maximum voltage, and steepness factor while also abrogating caffeine’s effects (Liu et al., 2021). This was compatible with RyR1-mediated Ca^2+^ release into a microdomain in the vicinity of both SR RyR1 and the T-tubular membrane Na_v_1.4 (Liu et al., 2021), permitting distinct local *in vivo* [Ca^2+^]_TSR_. This would increase with increased RyR1-mediated Ca^2+^ release but decrease with the SR Ca^2+^ depletion produced by the SERCA antagonist challenge despite their similar effects on bulk cytosolic [Ca^2+^]_i_ (Pagala and Taylor, 1998; Sekiguchi et al., 1999).

Similarly, murine cardiac muscle showed reduced *I*_Na_ when SR Ca^2+^ release was enhanced by Epac activation (King et al., 2013; Valli et al., 2018), or in the pro-arrhythmic gain of function RyR2-P2328S genetic modification associated with catecholaminergic polymorphic ventricular tachycardia, with implications for anti-arrhythmic therapy (Huang, 2017; Zhang et al., 2011; 2013). Cardiac CaMKII mutations similarly increase the risk of arrhythmias and heart failure (Swaminathan et al., 2012).

Third, K^+^ channel opening hyperpolarizes skeletal, cardiac, or smooth-cell membranes, similarly depressing their excitability. Intracellular C-termini of large conductance “Big K^+^” (BK) channels possess two regulatory Ca^2+^-binding sites (Sancho and Kyle, 2021; Zeng et al., 2005), conferring a μM local [Ca^2+^], additional to their voltage, sensitivity (Yang et al., 2015). Small-conductance (SK1, SK2, and SK3) K^+^ channels (Adelman et al., 2012; Weisbrod, 2020), although not voltage-sensitive, respond to intracellular Ca^2+^ including RyR-induced Ca^2+^ release (Neelands et al., 2001) through regulatory C-terminal CaM-binding domains. BK opening may reduce membrane excitability in exercising skeletal muscle (Allen et al., 2008) and modify cardiac sinoatrial node pacing (Meredith et al., 2014; Pineda et al., 2021). SK2 is implicated in pro-arrhythmic atrial and ventricular pathological situations (Yang et al., 2021; Lei et al., 2024; Li et al., 2023; Salvage et al., 2023). Fourth, regarding anion channels, Ca^2+^-activated Cl^−^ TMEM16A channels open in response to ∼600 nM free [Ca^2+^] with possible roles in cardiac (Horváth et al., 2016) and skeletal (Dayal et al., 2019) in addition to smooth muscle (Manoury et al., 2010).

Finally, regarding ion transporters, physiologically important in muscle membranes, Na^+^/Ca^2+^ exchangers (NCX) have been localised to both skeletal and cardiac muscle T-tubular membranes (Donoso and Hidalgo, 1989; Sacchetto et al., 1996). They can affect higher (10–50-fold) turnover rates than Ca^2+^-ATPase transporters, but their 10-fold lower Ca^2+^ affinity [*K*_d_∼1 μM; (Blaustein and Lederer, 1999)] is compatible with activity at the μM-level T-SR microdomain suggested here rather than nM-level bulk cytosolic [Ca^2+^].

Ca^2+^ microdomains also exerted potential reciprocal actions through their associated charge accumulation and direct surface membrane adsorption. Both potentially alter ∆*V*. The latter had previously been implicated in significant +30-mV ∆*V* alterations that could produce feedback effects from [Ca^2+^]_i_ elevations to 2 µM during excitation–contraction coupling (Pizarro et al., 1991). However, the present modelling using more realistic Ca^2+^–phospholipid affinities (Bers et al., 1985; Deplazes et al., 2021; McLaughlin et al., 1981; Melcrová et al., 2016) suggested that even both these effects together would produce <+7.4-mV voltage changes, unlikely to appreciably affect the activation or inactivation of surface membrane molecules, while not excluding the other mechanisms for “retrograde” regulation by RyR1 (Flucher, 2016; Huang et al., 2011).

This modelled T-SR junction Ca^2+^ microdomains could be important in clinical translational situations. Elevated skeletal muscle T-SR junctional microdomain [Ca^2+^] could inhibit tubular Na_v_1.4 function not only in normal sustained activity (Martin et al., 2003; Usher-Smith et al., 2007) but also in particular clinical skeletal myopathies (Dowling et al., 2014). The latter are exemplified by RyR1 mutation-related congenital myopathies or malignant hyperthermia susceptibility resulting from clinical loss-of-function, RyR1 Ca^2+^-binding site mutations (Witherspoon and Meilleur, 2016). Na_v_1.4 C-terminal EF hand-like domain mutations have been associated with a myotonic hyperexcitability disorder disrupting Ca^2+^-mediated inhibition of Na_v_1.4 function (Biswas et al., 2013; Horie et al., 2020). Abnormally increased myotube diameters and resting [Ca^2+^]_i_ and decreased RyR1-mediated Ca^2+^ release reflecting abnormal triad junction formation and maintenance are associated with a junctophilin (JP2) mutation (Woo et al., 2010). Transfection experiments reported reductions in muscle fatigue and improvement in contraction strength, following increased RyR1 phosphorylation produced by CaMKII overexpression (Flück et al., 2024).

In these examples, most of the microdomain Ca^2+^ accumulation would be likely attributable to RyR-mediated Ca^2+^ release. These are 1–2 orders of magnitude greater than early Cav1.1 or Cav1.2 tubular currents [skeletal muscle voltage clamp, *I*_Caf_ ∼25 μA cm^−2^ (Cota and Stefani, 1986); cardiomyocyte patch clamp *I*_CaL_ ∼10 pA pF^−1^ (Morinaga et al., 2019) yielding *J*_influx_ ∼ 8.64 × 10^−7^ and ∼6.91 × 10^−8^ mol m^−2^ s^−1^, respectively, assuming similar *C*_T_/*C*_s_ and *ξ*]. Larger skeletal muscle late *I*_Ca_ (80 μA/cm^−2^, yielding *J*_influx_ ∼ 2.76 × 10^−6^ mol m^−2^s^−1^) shows activation time courses (100 s of ms) too prolonged to drive excitation–contraction coupling (Sanchez and Stefani, 1978; Sánchez and Stefani, 1983).

The present study complements previous reports (Cannell and Allen, 1984) modelling overall Ca^2+^ diffusion over the entirety of an amphibian skeletal muscle myofibril half-sarcomere, following altered Ca^2+^ SR membrane permeability producing Ca^2+^ release. In their case, the latter was permitted to vary with a driving force dependent on consequent alterations in SR [Ca^2+^] and a varying eventual bulk cytosolic [Ca^2+^]. Furthermore, the terminal cisternae, longitudinal sarcoplasmic reticulum, and extramyofibrillar and myofibrillar spaces were each lumped into exchanging compartments, with appropriately localised Ca^2+^-binding proteins troponin, parvalbumin, and calsequestrin, possessing realistic binding kinetics. This diffusive, as opposed to full electrodiffusive, characterisation adopted free-Ca^2+^ diffusion coefficient values (7 × 10^−4^mm^2^ s^−1^) allowing for Ca^2+^ binding to cytoplasmic-binding sites. Here, we contrastingly specifically address electrodiffusive properties and the effect of cytosolic buffering of established potential physiological importance on these within an anatomically defined structure of dimensions corresponding to that of the skeletal muscle T-SR junction (Dulhunty, 2006; Franzini-Armstrong, 1970; Franzini-Armstrong, 1973). We thus examine its capacity for microdomain formation under a physically defined constant Ca^2+^ influx from the SR-Ca^2+^ store, eventually effluxing into a constant bulk cytosolic Ca^2+^. Further studies could incorporate into such a basic model the space occupied by an L-type Ca^2+^ channel and RyR molecules within the T-SR junction, including their geometrical and density distributions and their own rate and binding constants for Ca^2+^ binding, as previously applied for cardiac dyad junctions (Tanskanen et al., 2007). Incorporating such space-filling molecules would reduce the free volume of the T-SR space. They could then proceed to investigate contributions from Ca^2+^ adsorption onto enclosing membranes. Nevertheless, our present computations provide lower limiting, and useful, general indications of microdomain Ca^2+^ accumulation within a space corresponding to that of the T-SR geometry. For example, these findings demonstrate physiologically important elevated T-SR junctional Ca^2+^ levels comparable to the Ca^2+^-CaM-binding constant.

Closely apposed membranes potentially mediating localised Ca^2+^ signalling involving Ca^2+^-dependent proteins also occur widely in other cell types (Chang et al., 2017; Henkart et al., 1976). In smooth muscle, local Ca^2+^ release into SR–plasma membrane appositions (Devine et al., 1972) could increase repolarizing Ca^2+^-activated K^+^ channel activity even when cell-wide Ca^2+^ release activates MLCK, promoting contraction (Knot et al., 1998). Cerebellar Purkinje and hippocampal neurons (Wu et al., 2017) similarly signal using RyR-Ca^2+^ release channels (Kano et al., 1995; Kohda et al., 1995; Ouyang et al., 1997; Tedoldi et al., 2020), as recently implicated in *I*_Na_ modulation (Bertagna et al., 2024a; 2024b). Finally, in non-excitable thrombocytes, multiple 20–30-nm-diameter membrane invaginations in their open canalicular systems (OCSs) (Anand and Harper, 2020; Sage et al., 2013) form vacuolar structures apposed to membranes of the Ca^2+^-storing deep tubular system (DTS) structurally comparable with muscle T-SR junctions (Van Nispen Tot Pannerden et al., 2010). These would constitute an inositol trisphosphate receptor rather than RyR-mediated Ca^2+^ fluxes. Finally, both Ca^2+^ and Ca^2+^-CaM act on other signalling cascades involving soluble proteins: ∼µM Ca^2+^-CaM may also exert other cytosolic effects as on glyceraldehyde 3-phosphate dehydrogenase (Singh et al., 2004) or itself provide local signalling domains (Saucerman and Bers, 2012).

## Data Availability

The original contributions presented in the study are included in the article/Supplementary Material. Further inquiries can be directed to the corresponding author.
