# Supplementary material for: Nernst–Planck–Gaussian finite element modelling of Ca2+ electrodiffusion in amphibian striated muscle transverse tubule–sarcoplasmic reticular triadic junctional domains
Source: Front Physiol. 2024 Dec 5;15:1468333. doi: 10.3389/fphys.2024.1468333 (PMC11655509; doi:10.3389/fphys.2024.1468333)
Supplement: Supplementary file 1 [file DataSheet1.pdf]

Supplementary information: MATLAB Code

SPECIFY PARAMETERS

```
clear
clear global
tic
% Get the current date and time
CurrentDateAndTime = datestr(now,'yyyy-mm-dd HH-MM')
```

Geometry

```
% Radius of the T-SR junction (nm)
Disc_Radius =  110;
% Axial Distance (nm)
Disc_Width = 12;
% Maximum Mesh Tetrahedral Length (nm)
Mesh_Hmax = 15;
%surface charge must be run with a low Hmax to avoid noise due to irregular
%meshing
```

Definition of Constant Boundary Conditions

```
%Starting note : order of equations : [1,2,3,4] = [Cl-, Ca2+, K+, Donnan Protein]
% Flux Density across F1 (mol/nm2/s), the T-tubular membrane
F1_Flux = [0,0,0,0];
% Flux Density across F2 (mol/nm2/s), the SR-membrane
F2_Flux = [0, 3e-24, 0,0];
```

Constants

```
% Diffusion Coefficients (nm2/s)
D_Ca = 4e7;
D_K = 2e9;
D_Cl = 2e9;
D_CaM = 1.1e7;
D_Donnan = 1e7;
%CaM values
CaM = 24e-30; %CaM concentration (mol/nm3), taking into account the 4 binding sites
for Ca2+
Kd = 0.5e-30; %CaM Kd (mol/nm3)
% Faraday's (C/mol)
Faraday = 96485.309;
% Elementary Charge (C)
Elementary_Charge = 1.60217733e-19;
% Gas Constant (J/K/mol)
Gas_Constant = 8.314511;
% Absolute Temperature (K)
Abs_Temp_Celcius = 37;
Abs_Temp_Kelvin = 273.15 + Abs_Temp_Celcius;
% Permittivity (F/nm)
permittivity_free_space = 8.854187817e-21;
permittivity_cytoplasm = 80;
%Avogadro Constant (/mol)
Avogadro = 6.0221367e23;
```

```
%Specific Capacitance (F/nm2)
Sp_Capacitance = 1e-20;
% Generating Global variables for the coefficients of our PDEs which vary
% depending on current state of the solution
global a_coefficients
a_coefficients = Faraday / (Gas_Constant*Abs_Temp_Kelvin);
global omega_coefficients
omega_coefficients = Elementary_Charge /
(permittivity_free_space*permittivity_cytoplasm);
global f_coefficients
f_coefficients =
(Faraday*Faraday*Disc_Width)/(Gas_Constant*Abs_Temp_Kelvin*2*Sp_Capacitance);
global D_Ca;
global D_Cl;
global D_K;
global D_CaM;
global Kd;
global D_Donnan;
```

Exit length calculation

```
% Exit Length (nm)
Exit_Length = 9.2;
% Calculated co-efficient 'R' for Calcium Flux Density, passed onto F3
global R_Ca
R_Ca = D_Ca/Exit_Length;
```

Timings

```
% Modelling End Time (s)
T_End = 5e-4;
% Number of Time Points to Sample
T_Resolution = 1500;
% Calculate Sampling Interval
T_Spacing = T_End / T_Resolution;
```

MODEL PROGRAMMING

Geometry and Mesh

```
% Produce a cylindrical geometry with dimensions specified above
gm = multicylinder(Disc_Radius,Disc_Width);
% Generate geometry as assigned to PDE Model
model = createpde(4);
model.Geometry = gm;
% Generate a mesh on the geometry with properties described above
meshgeom = generateMesh(model,"Hmax",Mesh_Hmax);
% Copy a component of matrix meshgeom to noderet for figure production later
noderet=meshgeom.Nodes;
```

Definition of constant coefficients for Ca2+, counterions and Donnan Protein

```
%Specifies the Diffusion coefficients
Model_Coefficient_c = [D_Cl;D_Ca;D_K;D_Donnan];
% Specifying Coefficients
specifyCoefficients(model,"c",Model_Coefficient_c,"f",@fcoeffunction,"a",@acoeffunc
tion,"d",1,"m",0);
```

```
%The @ccoeffunction recruits the function involving CaM at the end of the
%code. To run with CaM, call @ccoeffunction instead  (run line below and
%silence line above)
%specifyCoefficients(model,"c",@ccoeffunction,"f",@fcoeffunction,"a",@acoeffunction
,"d",1,"m",0);
```

Application of Boundary and Initial Conditions for Ca2+, counterions and Donnan protein

```
% % Apply F1 flux across the T-tubular membrane
applyBoundaryCondition(model,"neumann","Face",1,"g",F1_Flux,"q",0);
% Apply F2 flux across the SR membrane
applyBoundaryCondition(model,"neumann","Face",2,"g",F2_Flux,"q",0);
% Apply F3 flux across the edge of the model. [1,3,4] specifies dirichlet
% conditions for Cl-, K+ and Donnan Protein while maintaiing Neumann and exit
length for
% Ca2+
applyBoundaryCondition(model,"mixed","Face",3,"u",[3e-27;1.42e-25;1.39e-
25],"EquationIndex",[1,3,4],"g",@F3_Ca_Flux);
%Define and apply initial ion concentrations (defined by resting
%concentrations)
u0 = [3e-27;5e-32;1.42e-25;1.39e-25];
setInitialConditions(model,u0);
```

SOLUTION

Run model

```
%tlist is the initial time : Sampling Interval : end time in seconds
tlist = 0:T_Spacing:T_End;
solution = solvepde(model,tlist);
%Convert Nodal solution into uM from mol/nm3, saved as sol
sol_row_one = solution.NodalSolution(:,1,:)*1e30;
sol_row_two = solution.NodalSolution(:,2,:)*1e30;
sol_row_three = solution.NodalSolution(:,3,:)*1e30;
Sol_time = solution.NodalSolution(:,:,:)*1e30;
timeForSolution = toc
```

MEMBRANE POTENTIAL CALCULATIONS (and example plots)

Calculating surface charge in presence and absence of Ca2+

```
%Identify points of the model corresponding to the T-tubular membrane
nidcoords4 = [0,0,12];
getClosestNode = @(p,x,y,z) min((p(1,:) - x).^2 + (p(2,:) - y).^2 + (p(3,:) -
z).^2);
[~,nid4]=getClosestNode(meshgeom.Nodes,nidcoords4(1),nidcoords4(2),nidcoords4(3));
u = sol_row_two(nid4,1501);


%Calculate Ca2+ binding to membrane phospholipids using 'Michaelis-Menten' kinetics
global CaM
```

```
global Kd
local_Kd = Kd * 1e30;
local_CaM = CaM * 1e30;
delta = (local_CaM+local_Kd+u).*(local_CaM+local_Kd+u) - 4*u*local_CaM;
x = ((local_CaM+local_Kd+u)-(delta.^0.5))/(2);
c=u-x;
PPL_total = 0.05;
sigma_total = PPL_total*Faraday/100000;
Kd_PPL = 100;
delta = (c+PPL_total+Kd_PPL)^2 - 4*c*PPL_total;
CaS = (c+PPL_total+Kd_PPL-sqrt(delta))/(2);
PPL = PPL_total - CaS;
sigma = PPL*Faraday/100000;


%Calculate the potential resulting from the surface charge in presence and
%absence of Ca2+
x = linspace(0,6e-9,100);
lambda = 1.5e-9;
T = Abs_Temp_Kelvin;
R = Gas_Constant;
e = permittivity_cytoplasm;
e0 = 8.9e-12;
b = 2*R*T*e*e0/(Faraday*sigma);
alpha = ((-2*b/lambda)+sqrt(4+(4*b^2)/lambda^2))/2
V=-2*R*T*log((1+alpha*exp(-x/lambda))./(1-alpha*exp(-x/lambda)))/Faraday;
b_total =2*R*T*e*e0/(Faraday*sigma_total);
alpha_total = ((-2*b_total/lambda)+sqrt(4+(4*b_total^2)/lambda^2))/2;
V_total=-2*R*T*log((1+alpha_total*exp(-x/lambda))./(1-alpha_total*exp(-
x/lambda)))/Faraday;


%Plot the graphs representing the potential resulting from surface charge


%define the layout
tiledlayout (2,1)


%first figure
nexttile
plot(x,V*1000,'r',"LineWidth",2.5)
hold on
plot(x,V_total*1000,'b',"LineWidth",2.5)
% Label the axes
xlabel('Distance from T-tubular membrane (nm)', 'FontSize', 16);
ylabel('Voltage (mV)', 'FontSize', 16);
xticks([0,1e-9,2e-9,3e-9,4e-9,5e-9,6e-9])
labels = {'With calcium', 'Without calcium'};
% Add a legend
legend(labels, 'Location', 'Best', 'FontSize', 14);
%Adjust line properties
set(gca, 'LineWidth', 1.5, 'FontSize', 16);
% Add a grid
grid on;
```

```
a = get(gca,'XTickLabel');
set(gca,'XTickLabel',a,'fontsize',16)
title(['Voltage generated by surface charge as' newline 'a function of distance to
the membrane'], 'FontSize', 20,'Units', 'normalized', 'Position', [0.5, 1.00, 0]);
hold off;


%second figure
nexttile
x2 = linspace(0,2e-10,100);
V=-2*R*T*log((1+alpha*exp(-x2/lambda))./(1-alpha*exp(-x2/lambda)))/Faraday;
V_total=-2*R*T*log((1+alpha_total*exp(-x2/lambda))./(1-alpha_total*exp(-
x2/lambda)))/Faraday;
plot(x2,V*1000,'r',"LineWidth",2.5)
hold on
plot(x2,V_total*1000,'b',"LineWidth",2.5)
% Label the axes
xlabel('Distance from T-tubular membrane (nm)', 'FontSize', 16);
ylabel('Voltage (mV)', 'FontSize', 16);
xticks([0e-10,0.5e-10,1e-10,1.5e-10,2e-10])
xticklabels({'0','0.05','0.1','0.15','0.2'})
labels = {'With calcium', 'Without calcium'};
% Add a legend
legend(labels, 'Location', 'Best', 'FontSize', 14);
%Adjust line properties
set(gca, 'LineWidth', 1.5, 'FontSize', 16);
% Add a grid
grid on;
a = get(gca,'XTickLabel');
set(gca,'XTickLabel',a,'fontsize',16)
title(['Voltage generated by surface charge as' newline 'a function of distance to
the membrane'], 'FontSize', 20,'Units', 'normalized', 'Position', [0.5, 1.00, 0]);
hold off;
```

Plot the change in membrane potential resulting from accumulation of Ca2+ in bulk T-SR cytosol

```
%Identify the appropriate points in the mesh
% Create a grid of Values
xradial = -Disc_Radius:1:Disc_Radius;
yradial = 0*ones(1,length(xradial));
zradial = yradial;


%Interpolate the solution at each time point for the graph for
% %spatio-temporal evolution across the solution grid
interpolate_radialplot1 =
interpolateSolution(solution,xradial,yradial,zradial,[2],17)*1e30;
interpolate_radialplot2 =
interpolateSolution(solution,xradial,yradial,zradial,[2],32)*1e30;
interpolate_radialplot3 =
interpolateSolution(solution,xradial,yradial,zradial,[2],63)*1e30;
```

```
interpolate_radialplot4 =
interpolateSolution(solution,xradial,yradial,zradial,[2],126)*1e30;
interpolate_radialplot5 =
interpolateSolution(solution,xradial,yradial,zradial,[2],251)*1e30;
interpolate_radialplot6 =
interpolateSolution(solution,xradial,yradial,zradial,[2],501)*1e30;
interpolate_radialplot7 =
interpolateSolution(solution,xradial,yradial,zradial,[2],1001)*1e30;
interpolate_radialplot8 =
interpolateSolution(solution,xradial,yradial,zradial,[2],1501)*1e30;


%Plot the voltage at each time point as a function of radial postion along
%the T-SR junction
figure;
plot(xradial,(((2*Faraday*(interpolate_radialplot8)*1e-3*Disc_Width*1e-9)/2e-
2)*1e3),"LineWidth",2.5)
hold on
plot(xradial,(((2*Faraday*(interpolate_radialplot7)*1e-3*Disc_Width*1e-9)/2e-
2)*1e3),"LineWidth",2.5)
plot(xradial,(((2*Faraday*(interpolate_radialplot6)*1e-3*Disc_Width*1e-9)/2e-
2)*1e3),"LineWidth",2.5)
plot(xradial,(((2*Faraday*(interpolate_radialplot5)*1e-3*Disc_Width*1e-9)/2e-
2)*1e3),"LineWidth",2.5)
plot(xradial,(((2*Faraday*(interpolate_radialplot4)*1e-3*Disc_Width*1e-9)/2e-
2)*1e3),"LineWidth",2.5)
plot(xradial,(((2*Faraday*(interpolate_radialplot3)*1e-3*Disc_Width*1e-9)/2e-
2)*1e3),"LineWidth",2.5)
plot(xradial,(((2*Faraday*(interpolate_radialplot2)*1e-3*Disc_Width*1e-9)/2e-
2)*1e3),"LineWidth",2.5)
plot(xradial,(((2*Faraday*(interpolate_radialplot1)*1e-3*Disc_Width*1e-9)/2e-
2)*1e3),"LineWidth",2.5)
%Note, the plot command above incorporates the Charge difference equation
%which allows to derive bulk-to-bulk transmembrane potential as a function
%of ion concentration
%Label the axes
xlabel('Radial distance (nm)', 'FontSize', 16);
YLabel = ylabel('Voltage (mV)', 'FontSize', 16);
xlim([-110,110])
xticks([-110,-55,0,55,110])
xticklabels({'-110','-55','0','55','110'})
labels = {'0.5ms','0.33ms','0.16ms','0.08ms','0.04ms','0.02ms','0.01ms','0ms'};
% Add a legend
legend(labels, 'Location', 'Best', 'FontSize', 14);
%Adjust line properties
set(gca, 'LineWidth', 1.5, 'FontSize', 16);
% Add a grid
grid on;
a = get(gca,'XTickLabel');
set(gca,'XTickLabel',a,'fontsize',16)
title(['Increase in membrane voltage' newline 'due to charge accumulation'],
'FontSize', 20,'Units', 'normalized', 'Position', [0.5, 1.00, 0]);
hold off;
```

DEFINITION OF NON-CONSTANT BCs AND COEFFICIENTS

Neumann BC for F3 (Ca2+ flux)

```
%F3 flux definition for model. This defines Ca2+ flux at F3
%(other ions are clamped by the Dirichlet condition defined in when
%specifying boundary conditions)
```

```
function a = F3_Ca_Flux (~,state)
global R_Ca
a = -R_Ca*state.u;
end
```

Defining CaM binding and updating the Ca2+ diffusion coefficient

```
%ccoeffunction : CaM, with finite CaM pool (does get depleted)
function a = ccoeffunction(region, state)
global D_Ca
global D_K
global D_Cl
global D_CaM
global Kd
global CaM
global D_Donnan
% Model CaM-Ca binding using 'Michaelis-Menten' kinetics
delta = (CaM+Kd+state.u(2))^2 - 4*state.u(2)*CaM;
x = (CaM+Kd+state.u(2)-sqrt(delta))/(state.u(2)*2); % x is the proportion of [Ca-
CaM] at a given node of the solution
%x = 1/(1+(Kd/CaM)); % this x value can be used to simulate an 'infinite'
%CaM pool
N_char = 4;
nr = numel(region.y);
a = zeros(N_char,nr);
a (1, :) = D_Cl*ones(1,nr);
a (2, :) = ((((1-x)*D_Ca + x*D_CaM)))*ones(1,nr);
a (3, :) = D_K*ones(1,nr);
a (4, :) = D_Donnan*ones(1,nr);
end
```

Define the non-constant coefficients for the Nernst-Plank equation

```
function a = acoeffunction(region, state)
global a_coefficients
global omega_coefficients
global D_Ca
global D_K
global D_Cl
global D_Donnan
N_char = 4;
nr = numel(region.y);
a = zeros(N_char,nr);
```

```
a(1,:) = D_Cl *a_coefficients * omega_coefficients * (state.u(1)-2*state.u(2)-
state.u(3)+state.u(4));
a(2,:) = -D_Ca * a_coefficients * omega_coefficients * (state.u(1)-2*state.u(2)-
state.u(3)+state.u(4));
a(3,:) = -D_K * a_coefficients * omega_coefficients *(state.u(1)-2*state.u(2)-
state.u(3)+state.u(4));
a(4,:) = -D_Donnan * a_coefficients * omega_coefficients * (state.u(1)-
2*state.u(2)-state.u(3)+state.u(4));
end



function f = fcoeffunction(region, state)
global f_coefficients
global D_Cl
global D_Ca
global D_K
global D_Donnan
NC = 4;
U_neg = gradient(state.u(1)+state.u(4));
U_pos = gradient(2*state.u(2)+state.u(3));
f = zeros(NC, length (region.x));
f(1,:) = -D_Cl * f_coefficients*dot((U_pos-U_neg),U_neg);
f(2,:) = D_Ca * f_coefficients*dot((U_pos-U_neg),U_pos);
f(3,:) = D_K * f_coefficients*dot((U_pos-U_neg),U_pos);
f(4,:) = D_Donnan * f_coefficients*dot((U_pos-U_neg),U_pos);
end
```
